# Epigenetic landscape reorganisation and reactivation of embryonic development genes are associated with malignancy in IDH-mutant astrocytoma

**DOI:** 10.1007/s00401-024-02811-0

**Published:** 2024-10-09

**Authors:** Santoesha A. Ghisai, Levi van Hijfte, Wies R. Vallentgoed, C. Mircea S. Tesileanu, Iris de Heer, Johan M. Kros, Marc Sanson, Thierry Gorlia, Wolfgang Wick, Michael A. Vogelbaum, Alba A. Brandes, Enrico Franceschi, Paul M. Clement, Anna K. Nowak, Vassilis Golfinopoulos, Martin J. van den Bent, Pim J. French, Youri Hoogstrate

**Affiliations:** 1 Department of Neurology, Erasmus MC Cancer Institute, Rotterdam, The Netherlands; 2 Department of Tumour Immunology, Erasmus MC Cancer Institute, Rotterdam, The Netherlands; 3 Department of Neurosurgery, Yale School of Medicine, New Haven, CT USA; 4 Department of Pathology, Erasmus MC Cancer Institute, Rotterdam, The Netherlands; 5 ICM Institute for Brain and Spinal Cords, Sorbonne University, Paris, France; 6 EORTC Headquarters, Brussels, Belgium; 7 Neurology Department, University Clinic Heidelberg, Heidelberg University & German Center, Heidelberg, Germany; 8 Neuro-Oncology Department, Moffitt Cancer Center, Tampa, FL USA; 9 Nervous System Medical Oncology Department, IRCCS Istituto delle Scienze Neurologiche di Bologna, Bologna, Italy; 10 Department of Oncology, Leuven Cancer Institute, KU Leuven, Leuven, Belgium; 11 Medical School, The University of Western Australia, Crawley, WA Australia

**Keywords:** Astrocytoma, RNA sequencing, DNA methylation, Molecular grading, IDH, HOX

## Abstract

Accurate grading of IDH-mutant gliomas defines patient prognosis and guides the treatment path. Histological grading is challenging, and aside from *CDKN2A/B* homozygous deletions in IDH-mutant astrocytomas, there are no other objective molecular markers used for grading. RNA-sequencing was conducted on primary IDH-mutant astrocytomas (*n* = 138) included in the prospective CATNON trial, which was performed to assess the prognostic effect of adjuvant and concurrent temozolomide. We integrated the RNA-sequencing data with matched DNA-methylation and NGS data. We also used multi-omics data from IDH-mutant astrocytomas included in the TCGA dataset and validated results on matched primary and recurrent samples from the GLASS-NL study. Since discrete classes do not adequately capture grading of these tumours, we utilised DNA-methylation profiles to generate a Continuous Grading Coefficient (CGC) based on classification scores from a CNS-tumour classifier. CGC was an independent predictor of survival outperforming current WHO-CNS5 and methylation-based classification. Our RNA-sequencing analysis revealed four distinct transcription clusters that were associated with (i) upregulation of cell cycling genes; (ii) downregulation of glial differentiation genes; (iii) upregulation of embryonic development genes (e.g. *HOX*, *PAX,* and *TBX*) and (iv) upregulation of extracellular matrix genes. The upregulation of embryonic development genes was associated with a specific increase of CpG island methylation near these genes. Higher grade IDH-mutant astrocytomas have DNA-methylation signatures that, on the RNA level, are associated with increased cell cycling, tumour cell de-differentiation and extracellular matrix remodelling. These combined molecular signatures can serve as an objective marker for grading of IDH-mutant astrocytomas.

**Supplementary Information:**

The online version contains supplementary material available at 10.1007/s00401-024-02811-0.

## Introduction

The fifth edition of the World Health Organisation (WHO) classification of central nervous system tumours (WHO CNS5) incorporates molecular markers to classify distinct CNS neoplasms [21]. Besides *CDKN2A/B* homozygous deletion (HD), there are no molecular markers that currently aid grading of IDH-mutant astrocytomas. Grading of CNS tumours is therefore mainly reliant on histopathological criteria. Grade 3 IDH-mutant astrocytomas are distinguished from grade 2 by the presence of focal or dispersed anaplasia and significant mitotic activity [21]. Marking these features in IDH-mutant astrocytomas can be challenging and subject to inter-observer variability [18, 40]. Several studies have explored the prognostic value of mitotic activity by assessing consecutive high-power fields, revealing worse outcomes for IDH-mutant astrocytomas in the absence of *CDKN2A/B* HD [18, 39]. However, others have not corroborated this [8, 29, 46]. Also, a specific threshold for designating WHO CNS5 grade 3 in IDH-mutant astrocytomas remains undefined [21]. However, precise tumour grading is crucial for improved patient surveillance and treatment, which was emphasised by results from the recently published INDIGO trial showing strong clinical efficacy of the IDH1/2 inhibitor vorasidenib in grade 2 IDH-mutant glioma [26].

Recent advances in molecular profiling resulted in the development of DNA methylation-based classification, which offers an objective alternative approach to histology-based grading [6, 28]. This is particularly valuable for distinguishing between tumours that are histologically similar but molecularly distinct. DNA-methylation-based classification has been integrated into WHO CNS5 as diagnostic criteria for several CNS-tumour types [42]. The “CNS tumour classifier” uses a large reference cohort of CNS tumours and predicts tumour (sub)classes that are determined on unsupervised methylation-intrinsic clusters (https://www.molecularneuropathology.org) [6]. In addition to classifying tumour types, the CNS-tumour classifier stratifies IDH-mutant astrocytomas into two subclasses, low- (A_IDH_LG) and high-grade (A_IDH_HG), with distinct prognoses [37].

In earlier work, we observed that the variation in DNAm profiles appears to be continuous and associated with tumour malignancy [37]. Here we used genome-wide DNA-methylation (DNAm) data from samples included in the multicentre CATNON randomised phase III clinical trial on anaplastic gliomas with absence of combined loss of the 1p and 19q chromosomal arms (1p/19q codeletion) [41] to construct a Continuous Grading Coefficient (CGC). We identified that increased malignancy is associated with glial dedifferentiation, upregulation of extracellular matrix (ECM) genes and increased expression of cell cycling genes. Also, whilst acknowledging continuity of tumour grading, our cut-off points for the CGC showed a more precise method of classification compared to WHO CNS5 and results from the ‘CNS tumour classifier’.

## Materials and methods

### Study cohort

We included subjects from the CATNON [41], TCGA [7] and GLASS-NL [1] studies. In the non-blinded randomised CATNON trial patients aged older than 18 years with newly diagnosed 1p/19q non-codeleted anaplastic gliomas and a WHO performance score of 0–2 were included [41]. This multicentre study included patients from 137 institutes across Australia, Europe and North America. The 2 × 2 factorial design compared radiotherapy alone or radiotherapy combined with adjuvant temozolomide to those receiving radiotherapy and concurrent temozolomide, or radiotherapy with both concurrent and adjuvant temozolomide (December 2007–September 2015). The primary endpoint was overall survival (OS), measured from the day of randomisation until death or last follow-up. More detailed information regarding endpoints, inclusion and exclusion criteria are described in the study protocol (PIII trial of anaplastic glioma without 1p/19q LOH (https://www.eortc.org)). In the present study, we specifically analysed the subset of IDH-mutant tumours from the CATNON trial (median follow-up time: 54.5 months, Supplementary Methods). The GLASS-NL study systematically investigated longitudinal changes in patients with an initial diagnosis of IDH-mutant astrocytoma who had undergone at least two resections with more than 6-month intervals in between [1]. This multicentre (*n* = 3) study included patients treated in The Netherlands. Treatment regimens in this cohort were heterogeneous and involved either radiotherapy or chemotherapy alone, or a combination of both, where progression was not consistently followed by surgical resection. Treatment regimens in the TCGA IDH-mutant astrocytoma cohort were not controlled by the study design and were based on standard clinical practises and personalised patient assessments. To explore mechanisms associated with increased malignancy prior to post-surgery treatment, we exclusively utilised molecular data from initial resections for the CATNON and TCGA datasets. The GLASS-NL dataset was partitioned into primary and recurrent subsets.

### Molecular and clinical data collection

For the CATNON dataset Copy Number Variation (CNV) data were extracted from earlier generated Infinium EPIC DNAm data [37], where HD and amplifications were defined according to previously described criteria [37]. *IDH1/2* mutation and 1p/19q codeletion status were obtained from previous work [37]. Detailed methodologies and protocols used for obtaining our previously generated Infinium 850k DNAm data are described in the Supplementary Methods.

For the GLASS-NL dataset, raw Infinium 850k DNAm and RNA-sequencing data were collected from the public data repository (DNAm: EGAS00001007546, RNA-sequencing: EGAS00001007551). *IDH1/2,* CNV and 1p/19q codeletion status were obtained from earlier work [44].

TCGA clinical, RNA-sequencing and Infinium 450k DNAm data were downloaded from the TCGA-LGG and TCGA-GBM projects [7] through the Genomic Data Commons (GDC) portal. CNV calls were obtained from cBioPortal (https://www.cbioportal.org). Annotations for the *IDH1/2* mutation and chromosome 1p/19q codeletion status were obtained from literature [7].

### CNS-tumour classifier

Methylation arrays were batch analysed for all datasets using the Heidelberg CNS-tumour classifier ("report_website_mnp_brain_v12.8_sample (Version 1.1)") [6]. To facilitate throughput of the excessive size of the datasets, classifications were batch executed, and results were batch-downloaded using our *pymnp* API (https://github.com/yhoogstrate/pymnp). The CGC were calculated from the predictBrain_v12.8 *_scores_cal.csv files:

$${\text{CGC}} = \ln \left( {\frac{{A_{{{\text{IDH}}_{{{\text{HG}}}} }} {\text{cal}}}}{{A_{{{\text{IDH}}_{{{\text{LG}}}} }} {\text{cal}}}}} \right)$$

Hereby *cal* refers to the calibrated score, which is determined through a probability calibration model. This model transforms the raw scores into probabilities reflecting the confidence in class assignment [6]. The CGC can be calculated using our publicly accessible, user-friendly tool (https://erasmusmc-neuro-oncology.github.io/Continuous_Grading_Classifier), which requires only the direct output from the CNS-tumour classifier. Methylation-based CNV estimations were obtained from the “cnvp_v5.2/*.bins.igv” files. We used a threshold of ± 0.10 log2-intensity value for loss and gain, where samples exceeding the cut-off value of 350 Mb were categorised as having a high CNV load [34].

### WHO CNS5 classification

All tumours included in this study were re-classified based on WHO CNS5 criteria, which included assessment of both *CDKN2A/B* HD and presence of microvascular proliferation and/or necrosis. Absence of 1p/19q codeletion was confirmed by methylation-based CNV estimates for 1p and 19q as determined by the conumee package integrated in the CNS-tumour classifier. For the CATNON dataset, two dedicated neuropathologists evaluated necrosis and microvascular proliferation at the central pathology review. For the GLASS-NL dataset, histopathological diagnosis and grade were reassessed by an experienced neuropathologist [44]. WHO CNS5 classification of TCGA samples was obtained from earlier work [47].

### CGC association with copy-number events

For each genomic bin from the “cnvp_v5.2/*.bins.igv” file (default bin size as implemented in the CNS-tumour classifier: 50,000 bp), we acquired a measure of malignancy per individual copy number event by conducting a Wilcoxon signed-rank test with False Discovery Rate (FDR)-adjustment on the CGC. Associations of the CGC with copy number events were calculated for the amplification and HD status independently. We used log2-intensity differences of + 0.35 and − 0.415 as cut-off for amplification and HD, as proposed in literature [34]. These tests were performed independently for each dataset, ensuring dataset-specific associations. Results were presented as log10(*p* values) for losses and − log10(*p* values) for gains.

### DNA-methylation analysis

Raw DNAm data of matched methylation and RNA samples were preprocessed as described earlier (Supplementary Methods) [37]. For each probe, the methylation intensity values from the methylated and unmethylated channels were converted to obtain beta-values (beta = methylated intensity/(methylated + unmethylated intensity)) [3]. These values were then transformed to *M*-values, which are logit-transformed beta-values, to improve homoscedasticity [9]. Linear regression modelling on the *M*-values was performed for Differential Methylation Probe (DMP) analysis. We excluded probes targeting the sex chromosomes and those lacking a UCSC RefGene annotation. Annotation data for probes were obtained from the original Illumina Infinium MethylationEPIC manifest file. DMP analysis was done using limma’s lmFit function for model fitting, and probe-wise fold changes of the *M*-values were estimated using empirical Bayes moderation (limma::eBayes) of the standard errors [30]. Probes with an absolute log2-transformed fold change (LFC) larger than 0.5 (FDR-adjusted *p* value < 0.01) were considered significantly differentially methylated. CpG sites were classified into those located within CpG islands, in the 2 kb regions adjacent to these islands (referred to as shores), and in the subsequent 2 kb regions beyond shores (known as shelves). In addition, CpG sites were annotated according to their genomic position, including regions near the transcriptional start site (TSS), within the gene body, and within the 5′ and 3′ untranslated regions (UTRs).

### Bulk RNA-sequencing

For this study, we RNA-sequenced material of tumour resections of the randomised phase 3 CATNON trial (NCT00626990). Tumour tissue samples from IDH-mutant astrocytomas in the CATNON dataset (*n* = 306) were obtained from adult patients through the European Organisation for Research and Treatment of Cancer (EORTC) study 26053-22054. RNA was extracted from formalin-fixed paraffin-embedded (FFPE) tissue blocks, stored at room temperature, using the RNeasy FFPE kit according to the manufacturer’s protocol (#73504, QIAGEN, Germany). Material was isolated by a technician not involved in the data analysis and only aware of pseudonymized storage identifiers. Library quality control included RNA integrity analysis using the Fragment Analyser (Agilent Technologies) and RNA concentration quantification with the Qubit fluorometer (Invitrogen). Samples with compromised integrity and RNA concentrations below 0.5 nM were excluded (*n* = 123). RNA-sequencing (*n* = 183 samples) was performed on an Illumina NovaSeq 6000 with 150 bp paired-end reads including Unique Molecular Identifier (UMI) tags (GenomeScan BV, Leiden, The Netherlands). Sample exclusion criteria and processing of FASTQ files into alignments and read counts are described in the Supplementary Methods. In total, 148 samples passed stringent quality control from which we only included first resections with matching methylation data (*n* = 138).

### Single-nucleus RNA-sequencing dataset and analysis

For single-nucleus RNA-sequencing (snRNA-seq) analysis of primary-recurrent astrocytoma samples, our in-house vanHijfte_2023 dataset was used. Initially, these samples were selected according to WHO CNS4 criteria, focussing on grade II/III primary tumours that progressed to grade IV recurrences. After review by a dedicated neuropathologist, we reclassified these samples based on WHO CNS5 criteria. Data preprocessing and normalisation were performed as described previously [44]. Briefly, nuclei doublets were filtered out using the *scDblFinder* package [11]. For every sample, a lower limit of unique reads was defined to exclude low complex measurements. In addition, nuclei with a mitochondrial read fraction of > 0.1 were removed. Next, data was normalised using the SCTransform V2 function from the SCTransform package [12]. All samples were integrated into one dataset using the reciprocal Principal Component Analysis (PCA) method as integrated in the Seurat package (v4) [32]. Cell types were annotated using markers for normal cell types and malignant transcriptomic cell states [25, 27, 45]. Enrichment of marker gene sets was assessed using the gene function described previously [27]. In short, for a gene set G, a reference gene set R was created. First, all genes were separated into 30 equal-sized bins according to expression level. Next, for every gene in G, 100 random genes from its corresponding bin were added to R, resulting in R being 100× larger than G. The difference in average expression for G and R is calculated for every nucleus to provide an enrichment score.

### mRNA expression analysis

All RNA-sequencing datasets underwent consistent processing of raw mRNA counts. Lowly expressed genes with an average read count of less than three reads per sample were omitted. Also, only genes with *gencodev34* gene type annotation "protein coding" and "lncRNA" were included. DESeq2 [22] was used to estimate variance-stabilised transformed (VST) normalised read counts for each dataset separately. Differential Gene Regression (DGR) analysis was performed using the Wald test [22] where the CGC was used as continuous condition. Batch correction was not performed, since CATNON samples were sequenced in a single batch and no clustering indicative of batch effects was observed. Genes with an absolute LFC higher than 0.5 and FDR-adjusted *p* value smaller than 0.01 were considered significantly differentially expressed. Recursive-based correlation clustering on the differentially expressed genes was performed using the recursiveCorPlot package [14, 15]. Clusters containing 50 genes or fewer were excluded from further analyses.

### Correlation between RNA expression and DNA-methylation

Probe-level *M*-values were transformed to gene-level *M*-values by computing the median *M*-values of all probes linked with a gene as defined by the UCSC RefGene annotation. Genes also present in the processed RNA-sequencing data were then included. The overall correlation between the DMP (log2FoldChange *M*-value) and DGR (log2FoldChange normalised expression value) analyses were visualised by comparing the *z*-statistics per gene (log2FoldChange/standard error log2FoldChange).

### Immunohistochemistry staining and analysis

To determine protein expression, automated immunohistochemistry (IHC) was performed using the Ventana Benchmark ULTRA system (Ventana Medical Systems Inc.). Sequential 4 µm thick FFPE sections were subjected to deparaffinization followed by heat-induced antigen retrieval with CC1 (#950-500, Ventana) for 32 min. Sections were then incubated at 37 °C with HOXD10 (1:2500 dilution, rabbit polyclonal, Invitrogen) and Ki-67 (1.04 µg/ml, rabbit monoclonal, Ventana clone 30-9) antibodies. Incubation was followed by detection carried out using the OptiView detection kit (#760-700, Ventana), with 3,3′-diaminobenzidine (DAB) as the chromogen. Hematoxylin II counterstaining was applied for 8 min, followed by a blue colouring reagent for an additional 8 min, according to the manufacturer’s instructions. Control tissues were placed on adjacent parts of consecutive slides.

For Ki-67 analysis specifically, digitised slides were analysed in QuPath version 0.4.4 using the Brightfield H-DAB setting. Regions of interest (ROIs) were selected, excluding artefacts. Nuclei were detected using the StarDist 2D deep-learning based model (dsb2018_heavy_augment.pb). An artificial neural network (ANN_MLP) classifier, implemented in QuPath, was trained to distinguish Ki-67 positive from negative cells.

#### Statistical analysis and visualisation

All statistical analyses were performed in R programming language (v.4.2.2). Survival analysis was performed using the *survival* package (v3.5-7). We examined OS as clinical endpoint for all survival analyses. Overall survival probabilities were calculated using the Kaplan–Meier estimator and log-rank tests were used to compare survival curves. Cox proportional hazard (PH) regression modelling was performed to determine hazard ratios (HR) for univariable and multivariable analyses, where significance was assessed using the likelihood ratio test. For multivariable analyses, comparisons were made against markers already included in WHO CNS5 (*CDKN2A/B* HD and microvascular proliferation/necrosis). Cut-off points for the CGC were determined unsupervised with respect to clinical end points by assessing the rate of change of $A_{{{\text{IDH}}_{{{\text{HG}}}} }} {\text{cal}}$ per unit increase in CGC. The CGC-based “medium-grade” subgroup comprised 95% of the total cumulative rate. Cut-off values were first defined on CATNON and independently validated on TCGA and GLASS-NL. Gene set enrichment analysis (GSEA) was performed using the *clusterProfiler* package (v4.6.2). Molecular Signatures Database (MSigDB) human gene set collections (C2 and C5) were extracted using the *msigdbr* package (v.7.5.1) [35]. Plots were generated with the *tidyverse* package (v2.0.0). Survival forest plots of CoxPH models were visualised using the *survminer* package (v0.4.9)*.*

## Results

### Sample cohort

To investigate biological associations underlying malignancy of IDH-mutant astrocytomas, four large multi-domain and multi-centre omics datasets were leveraged: CATNON, TCGA, GLASS-NL primary (GLASS-NL-P) and GLASS-NL recurrent (GLASS-NL-R) [1, 7, 41]. We obtained DNAm (CATNON: *n* = 430, TCGA: *n* = 256, GLASS-NL-P: *n* = 98, GLASS-NL-R: *n* = 137) and DNA-sequencing (CATNON: *n* = 424, TCGA: *n* = 253, GLASS-NL-P: *n* = 97, GLASS-NL-R: *n* = 133) data for each of the studies. RNA-sequencing of primary IDH-mutant astrocytomas included in the CATNON trial was successfully performed for 138 samples and extended with transcriptomic data from the TCGA (*n* = 247), GLASS-NL-P (*n* = 65) and GLASS-NL-R (*n* = 102) datasets.

The median age at diagnosis was significantly lower in the GLASS-NL (32 [18–70]) dataset in comparison to both CATNON (37 [18–82], *p* = 5.92e−07) and TCGA (37 [14–74], *p* = 9.95e−06). The percentage of primary samples with WHO CNS5 grade 4 was higher in CATNON (13%), compared to GLASS-NL (9%) and TCGA (8%).

### Continuous grading coefficient as a measure for grading/malignancy

For all datasets, methylation data were uploaded into the DNAm-based CNS-tumour classifier for classification and copy number variation estimations. Three samples from the TCGA dataset exhibited 1p/19q codeletion (TCGA-CS-5394, TCGA-FG-7637, TCGA-VM-A8CA) and were therefore removed from all analyses (Supplementary Fig. 1a).

The vast majority of the samples were classified as IDH-mutant astrocytoma (CATNON: 412/430, TCGA: 244/253, GLASS-NL-P: 93/98, GLASS-NL-R: 116/137). The highest fraction of A_IDH_HG samples were observed in the CATNON and GLASS-NL-R datasets (CATNON: 98/412, TCGA: 25/244, GLASS-NL-P: 4/93, GLASS-NL-R: 45/116) (Supplementary Fig. 1a). This aligns with the inclusion criteria of CATNON and the higher proportion of progressed recurrent IDH-mutant astrocytoma in GLASS-NL [44].

Samples not classified as A_IDH_LG/A_IDH_HG were most often classified as oligodendroglioma (O_IDH, *n* = 11) and oligosarcoma (OLIGOSARC_IDH, *n* = 15) (Supplementary Fig. 1a/b). Oligosarcoma is not recognised as a distinct tumour type by WHO CNS5, but represents oligodendroglioma with mixed oligodendroglial and sarcomatous morphology [31, 36]. Oligosarcoma forms a unique distinct methylation class that has been incorporated in the latest version (v12.8) of the CNS-tumour classifier [36]. Samples classified as oligosarcoma did not harbour 1p/19q codeletion and had a lower tumour-purity estimate compared to other IDH-mutant methylation subclasses (Supplementary Fig. 1c, Supplementary Methods). TERT promoter mutations were absent in TCGA and CATNON samples classified as oligosarcoma [36]. Moreover, four GLASS-NL cases, classified as astrocytoma (A_IDH_LG) in the first resection, were later classified as oligosarcoma in their matched recurrent samples. All samples classified as oligosarcoma harboured astrocytoma-like features, including ATRX and/or TP53 mutations.

**Fig. 1:**
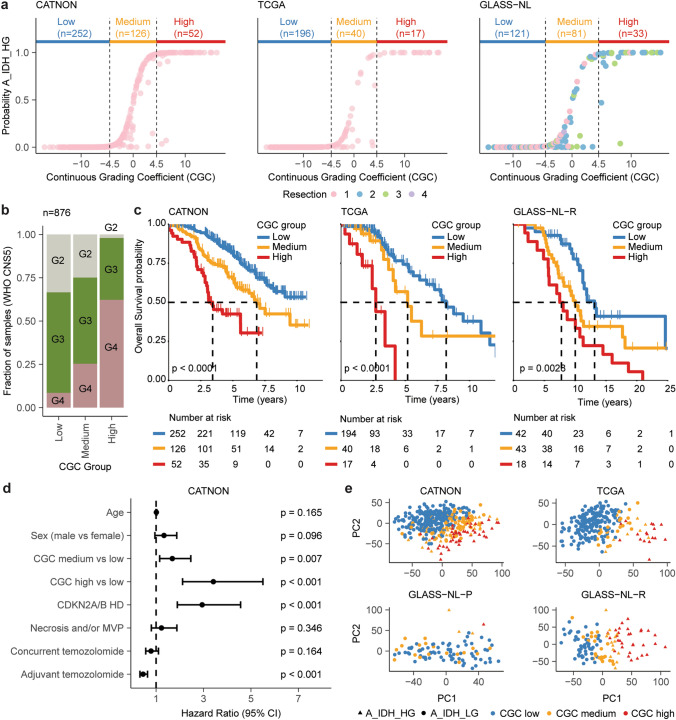
Evaluation of the three DNAm-based CGC-based subgroups (low, medium, high) across CATNON, TCGA and GLASS-NL. **a** Cut-off values for the three CGC groups (low: CGC < − 4.5, medium: CGC [− 4.5, 4.5], high: CGC > 4.5) which were determined based on the association between the CNS-tumour classifier probability score (A_IDH_HG) and the CGC. **b** Bar plot depicting the fraction of samples within each CGC subgroup according to WHO CNS5. **c** Kaplan–Meier overall survival curves stratified by CGC subgroup. *p* values were determined by log-rank test. **d** Survival forest plots showing the results of Cox proportional hazard models for CGC subgroups corrected for age, sex, WHO CNS5 criteria (*CDKN2A/B* HD and histological features) and treatment arms according to the CATNON trial. **e** Unsupervised principal component 1 and 2 of DNAm data demonstrates spatial segregation of IDH-mutant astrocytomas. CNS-tumour classifier subtype assignment (A_IDH_LG/A_IDH_HG) and CGC subgroups are indicated

We utilised the calibrated classification probabilities derived from the CNS-tumour classifier to generate a DNAm-based continuous grading coefficient (CGC). Formally, the CGC is calculated as the natural logarithm of the calibrated classification probabilities between A_IDH_LG and A_IDH_HG. We then defined three revised astrocytoma subtype classes (low: CGC < − 4.5, medium: CGC [− 4.5 to 4.5], high: CGC > 4.5) based on the relation between the CGC and the calibrated probability scores from the CNS-tumour classifier in CATNON (Fig. 1a). Clinical (age, sex, treatment), histological (necrosis and/or microvascular proliferation) and molecular (CNS-tumour classifier class, CNV load and *CDKN2A/B* HD) characteristics of patients within each CGC class are presented in Supplementary Table 1.

As may be expected, CGC class was positively associated with the number of samples being classified as WHO CNS5 grade 4 (CATNON: *p* < 0.0001, TCGA: *p* < 0.0001, GLASS-NL: *p* < 0.0001, Fisher Exact Test, Fig. 1b, Supplementary Table 1). However, a substantial proportion of WHO CNS5 grade 4 tumours were present in the CGC low and medium subgroups. Similarly, not all CGC-high tumours were WHO CNS5 grade 4. Our CGC subgroups were strongly associated with OS: median OS in CGC-low not reached (95% CI [8.2–not reached]), with CGC-medium 6.9 years (95% CI [5.7–not reached]) and CGC-high 3.4 years (95% CI [3.0–not reached]). All three CGC subgroups showed significantly different OS (CGC-medium vs CGC-low: HR 1.90, 95% CI [1.31–2.76]; *p* < 0.001, CGC-medium vs CGC-high HR: 2.12 95% CI [1.33–3.36]; *p* = 0.001). Importantly, CGC cut-off values determined on CATNON also showed prognostic significance in TCGA (*p* < 0.0001) and GLASS-NL-R (*p* < 0.005) (Fig. 1c). In these independent datasets, the CGC subgroups were significantly associated with CDKN2A/B HD (*p* < 0.0001, Fisher Exact Test, Supplementary Table 1).

Multivariable Cox PH-regression analysis on CATNON showed that the CGC subgroups were an independent prognostic factor (CGC-medium vs CGC-low: HR: 1.68, 95% CI [1.15–2.46] and CGC-high vs CGC-medium: HR: 3.42, 95% CI [2.12–5.51]) when adjusted for age, sex, *CDKN2A/B* HD, necrosis and/or microvascular proliferation and treatment with adjuvant/concurrent temozolomide (Fig. 1d). The CGC was also significantly associated with survival in multivariate analyses, outperforming WHO CNS5 in both the TCGA and GLASS-NL datasets (Supplementary Fig. 2, TCGA: *p* = 0.003, GLASS-NL: *p* = 0.007, Wald test).

High CNV load is associated with worse outcome in IDH-mutant astrocytoma [34, 37]. We found that CGC groups were significantly associated with CNV load in all datasets (*p* < 0.0001, Fisher Exact Test, Supplementary Table 1). CNV load was also associated with OS in a univariate analysis (CATNON: HR: 1.42 95% CI [1.02–1.97]; *p* = 0.038, TCGA: HR: 2.42 95% CI [1.38–4.23]; *p* = 0.0021, GLASS-NL-R: HR: 2.39 95% CI [1.45–3.96]; *p* < 0.001). Earlier work reported worse survival for gliomas with heterozygous deletion of *CDKN2A/B* [17]. To further investigate this, we manually assessed CNV profiles in the CATNON dataset, blinded to all other clinical and molecular parameters. This manual inspection was necessitated by the difficulty in distinguishing heterozygous deletion using conservative cut-off values. We identified 7 cases with a heterozygous deletion of *CDKN2A/B*. Patients without a deletion had a median OS of 9.45 years (95% CI [7.52–not reached]), whilst those with a heterozygous deletion showed a median OS of 4.46 years (95% CI [2.97–not reached]). Patients with *CDKN2A/B* HD conferred worse survival, with a median OS of 3.11 years (95% CI [2.83–5.60]). However, limited by the insufficient sample size, there was no significant difference in survival between patients with a heterozygous deletion and those with wild-type *CDKN2A/B* (*p* = 0.12, log-rank test).

We wondered to what extent our CGC captured the overall variability of DNAm profiles in our samples. To test this, we summarised the global DNAm profile of samples classified as IDH-mutant astrocytoma by PCA on the 10,000 most variable probes. Summarising the DNAm profile by our three CGC subgroups captured the global methylation profile better compared to A_IDH_LG and A_IDH_HG (Fig. 1e).

**Fig. 2:**
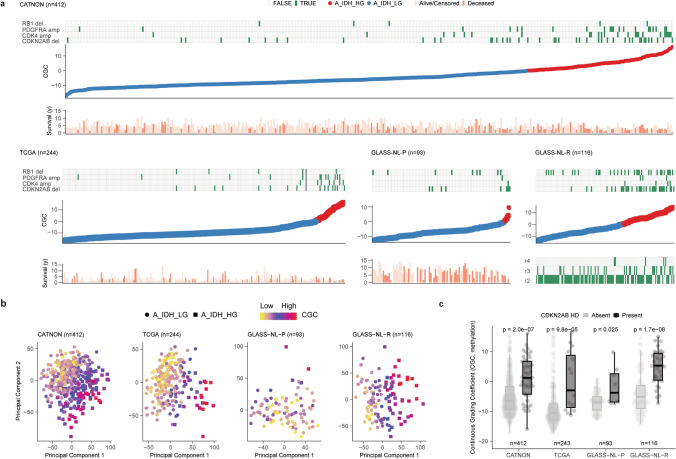
Continuous Grading Coefficient (CGC) as a tool to study malignancy in IDH-mutant astrocytoma.** a** Samples of individual cohorts (CATNON, TCGA, GLASS-NL) ranked according to their CGC. As can be seen, specific copy number of events (RB1 HD, PDGFRA amplification, CDK4 amplification and CDKN2A/B HD) and overall survival were correlated with the CGC. Samples are coloured based on astrocytoma subtype assignment (blue: A_IDH_LG, red: A_IDH_HG). **b** Unsupervised PC1 and PC2 on the DNAm data showing spatial segregation of samples classified as IDH-mutant astrocytoma. **c** Distribution of the CGC based on *CDKN2A/B* HD status across the different datasets. Samples with *CDKN2A/B* HD showed a significantly higher CGC. *p* values determined by Wilcoxon signed-rank test

The CGC for samples in the CATNON, TCGA, GLASS-NL-P, GLASS-NL-R datasets and common copy number events are illustrated in Fig. 2a. We observed that the spatial distribution of methylation profiles along PC1 and PC2 was captured more effectively by the CGC for all datasets, emphasising the efficacy of a continuous approach (Fig. 2b). High CGC values were not restricted to *CDKN2A/B* HD tumours alone (Fig. 2a/c) and we next explored associations between the CGC and copy number events (Supplementary Fig. 3). In addition to *CDKN2A/B* HD (CATNON: *p* = 6.60e−07, TCGA: *p* = 6.78e−05, GLASS-NL: *p* = 1.88e−08), we also found associations with *SMARCA2* HD (CATNON: *p* = 7.70e−05, TCGA: *p* = 3.62e−04, GLASS-NL: *p* = 6.68e−04), *RB1* HD (CATNON: *p* = 6.40e−04, TCGA: *p* = 0.033, GLASS-NL: *p* = 0.019), *PTEN* HD (CATNON: *p* = 2.73e−06, TCGA: *p* = 0.12, GLASS-NL: *p* = 1.69e−03), *CDK4* amplification (CATNON: *p* = 5.15e−05, TCGA: *p* = 0.016, GLASS-NL: *p* = 0.046) and *PDGFRA* amplification (CATNON: *p* = 5.15e−05, TCGA: *p* = 0.019, GLASS-NL: *p* = 0.024).

### Global decrease in DNA-methylation and hypermethylation of CpG islands associates with continuous grading coefficient

**Fig. 3:**
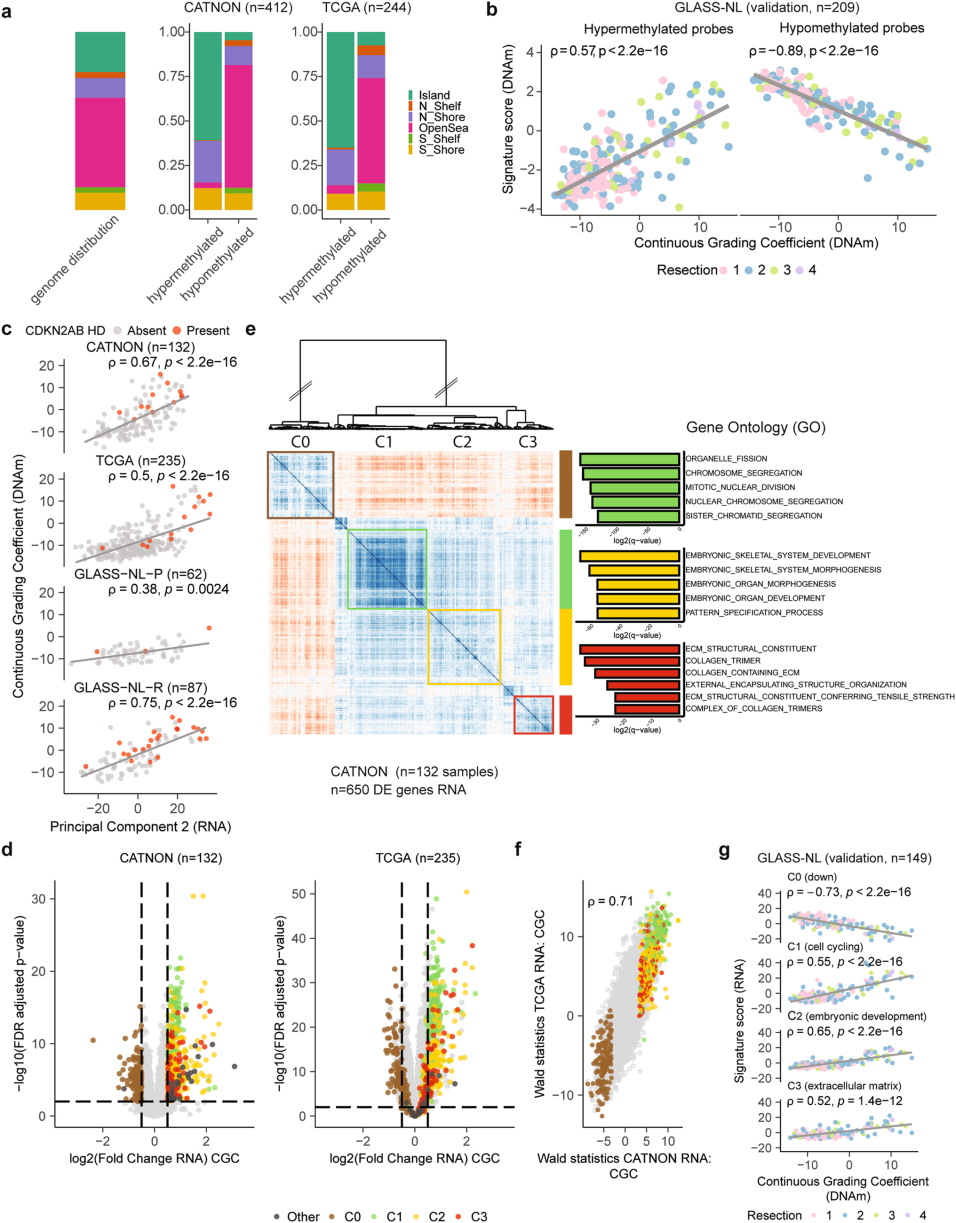
Supervised DNAm and RNA analysis on CATNON and TCGA with validation on the GLASS-NL dataset identifies gene clusters associated with malignancy. **a** Distribution of probes belonging to CpG islands, shelfs (N_Shelf, S_Shelf), shores (N_Shore, N_Shelf) and the open sea across all hypomethylated and hypermethylated probes. Distributions are displayed separately for the DMPs resulting from independent analyses conducted on CATNON (850k chip) and TCGA (450k chip). Genome-wide distribution of all probes is shown on the left as a reference. *p* values were determined by Fisher’s Exact Test. **b** Correlation between the DNAm-based signature scores of the hypermethylated and hypomethylated probes with the CGC in GLASS-NL (850k chip). **c** Spearman correlation between CGC and unsupervised PC2 of the transcriptomic data in all datasets. **d** Volcano plots showing the per-gene RNA log2FoldChange on the CGC and the corresponding FDR adjusted *p* value for both the CATNON and TCGA datasets. C0, C1, C2, and C3 genes are indicated.** e** Recursive-based correlation plot on VST expression of the differentially expressed genes in CATNON. Three upregulated (C1: green, C2: yellow, C3: red) and one downregulated (C0: brown) cluster were distinguished. Gene Ontology enrichment analysis resulted in significant hits for three clusters (C1: cell cycling, C2: embryonic development, C3: ECM).** f** Correlation between the DGE tests on the CATNON and TCGA datasets. For each gene the log2FoldChange divided by its standard error (Wald statistics) is indicated. **g** Spearman correlation between the RNA-based signature scores for C0, C1, C2, and C3 and the CGC in GLASS-NL.

To elucidate which CpG sites associate with malignant transformation as defined by the CNS-tumour classifier, we applied DMP analysis on the CGC using linear regression modelling for each dataset separately. In total, 8% of all tested probes were differentially methylated in the CATNON dataset and 8% in the TCGA dataset. In both datasets, the vast majority of differentially methylated probes were all hypomethylated in higher grade malignancies (CATNON: 99%, TCGA: 98%). Interestingly, although the vast majority of CpG sites had decreased methylation levels, those present on CpG islands were more often hypermethylated than hypomethylated (CATNON: *p* < 2.2e−16, TCGA: *p* < 2.2e−16, Fig. 3a, Supplementary Table 2). This observation is further supported by the finding that a larger fraction of hypermethylated probes belonged to the TSS200 region (Supplementary Fig. 4). Hypomethylated probes were typically found in the open sea, located more than 4 kb away from CpG islands. Focussing on DMP present in both the 450k (TCGA) and 850k (CATNON) array, we found that ~ 60% of the hypermethylated probes were shared.

We subsequently validated the overlapping DMPs identified in the TCGA and CATNON datasets on GLASS-NL. The median *M*-value of the overlapping hypermethylated (*n* = 149) and hypomethylated (*n* = 12,708) probes both showed a correlation with the CGC and resection number in the GLASS-NL cohort (hypermethylated: *ρ* = 0.57, hypomethylated: *ρ* = − 0.89, Fig. 3b). These correlations may be explained by an increased malignancy over time [44].

### Distinct transcriptional features are associated with continuous grading coefficient

We included RNA-sequencing data of IDH-mutant 1p/19q non-codeleted CATNON and TCGA samples and findings were further validated on GLASS-NL-P and GLASS-NL-R. First, we conducted unsupervised PCA on the 1000 most variably expressed genes for each dataset independently. In all datasets, the primary source of variation (PC1) was likely associated with tumour purity as demonstrated by the expression of neuronal genes, with neuron marker genes like *SLC12A5*, *TMEM130* and *SV2B* ranking among the top 50 contributors [25]. To confirm that the neuronal marker signature is associated with tumour purity, we used the top 50 most contributing genes of PC1 from each dataset and calculated the enrichment score for these genes. We projected this enrichment score onto our snRNA-seq data. These data indeed revealed that expression was primarily derived from neurons (Supplementary Fig. 5a/b). PC2 was however strongly associated with the DNAm-based CGC (CATNON: *ρ* = 0.67, TCGA: *ρ* = 0.50, GLASS-NL-P: *ρ* = 0.38, GLASS-NL-R: *ρ* = 0.75, Fig. 3c).

Gene-level differential regression analysis on samples from the CATNON and TCGA was then performed to find genes associated to the CGC. To ensure we are mainly investigating tumour-intrinsic signals, we estimated tumour purity using two methods and evaluated the outcomes by comparing their correlation with RNA expression levels of neuron markers (Supplement Methods, Supplementary Fig. 6a). We did not observe a significant difference in tumour purity based on *CDKN2A/B* HD status (CATNON: *p* = 0.46, TCGA: *p* = 0.39, Supplementary Fig. 6b). Also, there was no clear dependency of tumour purity on the outcome of the DGR analysis (CATNON: *ρ* = − 0.23, TCGA: *ρ* = 0.28, Supplementary Fig. 6c). Therefore no additional purity corrections were performed.

DGR on the CGC revealed a total of 650 differentially expressed genes in the CATNON dataset (Supplementary Table 2). The differential gene expression was skewed, with a higher proportion (77%) of genes showing increased expression in samples with a higher CGC (Fig. 3d). We then performed recursive correlation-based clustering [14] and identified four distinct gene clusters (Fig. 3e, C0/C1/C2/C3). The clusters that showed increased expression in more malignant samples (high CGC) were associated with specific biological functions as determined by gene ontology: C1 (cell cycling, *n* = 175), C2 (embryonic development, *n* = 176) and C3 (ECM, *n* = 97) (Fig. 3e). C0 (*n* = 149), which contained genes with a decreased expression in more malignant samples, could not be attributed to a biological function. DGR analysis on the TCGA dataset resulted in 667 differentially expressed genes (Fig. 3d), with a strong overlap with those identified with the CATNON analysis (58%). Also, the majority of the differentially expressed genes were upregulated (72%). Despite the differences in study designs, we observed a strong correlation across both datasets in outcome of the tests performed (*ρ* = 0.71, Fig. 3f).

Subsequently, we further examined the genes present within each of the transcriptional clusters. C1 contained genes associated with histones (e.g. *H3C2*, *H2BC9*, *H2BC11*), transcription factors (e.g. *E2F1*, *E2F8*, *FOXM1*), kinesins (e.g. *KIF14*, *KIF15*, *KIFC1*), DNA replication (e.g. *RAD51*, *EXO1*, *CENPK*) and cell cycle control (e.g. *CHEK1*, *CCNB1/2*, *CDK6*). Of note, established markers for cell cycling, such as *MKI67* and *TOP2A* were found. C2 contained developmental transcription factor genes, such as members of the *HOX* (*n* = 18), *PAX* (*n* = 3) and *TBX* (*n* = 2) gene family. Within C3, genes associated with ECM formation were found, such as *COL1A1* and *COL1A2*. On the contrary, C0 comprised many long non-coding RNA genes (*n* = 81). We compared expression levels of C0 genes amongst purified human CNS cell types (Supplementary Methods, Supplementary Fig. 7), and observed high expression levels in mature astrocytes as compared to foetal astrocytes (*p* < 0.001). Conversely, upregulated genes (C1-C3) were higher expressed in foetal astrocytes (*p* < 0.001). These combined findings suggest tumour dedifferentiation during malignant transformation.

We further examined genes present in our transcriptional clusters on protein level by IHC using DAB staining in CGC-low/medium (*n* = 5) and CGC-high (*n* = 5) samples. Quantification of Ki-67-positive cells revealed a significantly higher fraction of positive cells in the CGC-high group (Supplementary Fig. 8a, *p* = 0.03). *HOXD10* was also selected for IHC since it emerged as one of the top hits in the DGR analysis. Whilst HOXD10-positive cells were detected, the staining exhibited high background signal and was predominantly cytoplasmic, which complicated accurate quantification. Nonetheless, HOXD10 was more prominent in CGC-high regions compared to CGC-low regions (Supplementary Fig. 8a).

PCA was conducted individually for each cluster (C0–C3) to derive a per tumour-sample representative value. This value summarised the expression pattern of genes within the respective cluster, as represented by PC1. Of note, the differentially expressed genes used to define this RNA signature were identified solely based on results from CATNON. When these values were calculated on the GLASS-NL dataset, we found correlations between the CGC and all RNA signature scores (C0: *ρ* = − 0.73, C1: *ρ* = 0.55, C2: *ρ* = 0.65 and C3: *ρ* = 0.52, Fig. 3g).

**Fig. 4:**
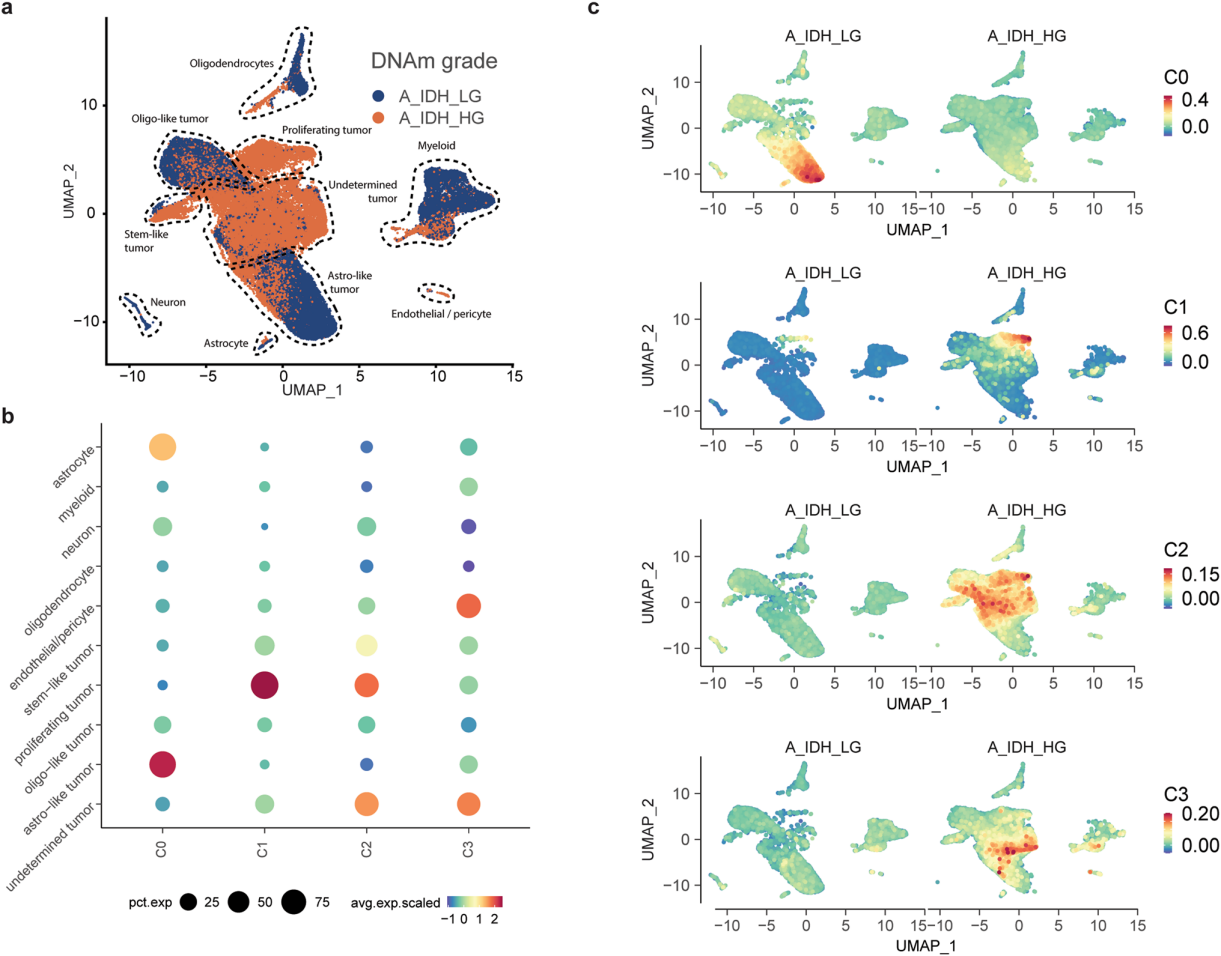
Single-nucleus RNA-sequencing (A_IDH_LG: *n* = 3, A_IDH_HG: *n* = 3) validates bulk DGE results and shows enrichment of gene clusters in select cell subpopulations. **a** UMAP projection illustrating cell-type annotations for all tumours combined. **b** Dotplot displaying the enrichment score of each of the bulk RNA-sequencing clusters (C0, C1, C2, and C3) identified in our DGR analysis. **c** UMAP projection showing enrichment scores of the downregulated (C0), cell cycling (C1), embryonic development (C2), and ECM (C3) clusters for A_IDH_LG and A_IDH_HG separately

We utilised our snRNA-seq dataset (vanHijfte_2023), which included six matched primary (A_IDH_LG) and recurrent (A_IDH_HG) IDH-mutant astrocytoma samples from three patients (Fig. 4a). Uniform Manifold Approximation and Projection (UMAP) showed distinct cell types and the previously reported tumour cell states (astro-like, oligo-like and stem-like, Fig. 4a, Supplementary Fig. 5a). Interestingly, at tumour recurrence/high-grade, the oligo-like and astro-like cell states were less pronounced and a substantial tumour cell cluster could not reliably be defined using our marker gene set. We then overlaid our transcriptional signatures (C0-C3) with this dataset and found that C0, C1 and C2 were predominantly expressed in tumour cells (Fig. 4b/c). More specifically, C0 and C1 showed high expression in astro-like and proliferating tumour cells respectively [16, 45]. Genes from C3 were equally expressed by endothelial cells/pericytes and undetermined tumour cells (Fig. 4b/c). In line with our RNA-sequencing results, expression of C0 genes decreased and C1/C2/C3 increased during malignant transformation. In summary, our data suggest that malignant transformation of IDH-mutant astrocytoma is associated with an upregulation of cell cycling, embryonic development and ECM genes. This was accompanied by decreased expression of astro-like state genes.

### Hypermethylation and upregulation of embryonic development genes in more malignant IDH-mutant astrocytoma

**Fig. 5:**
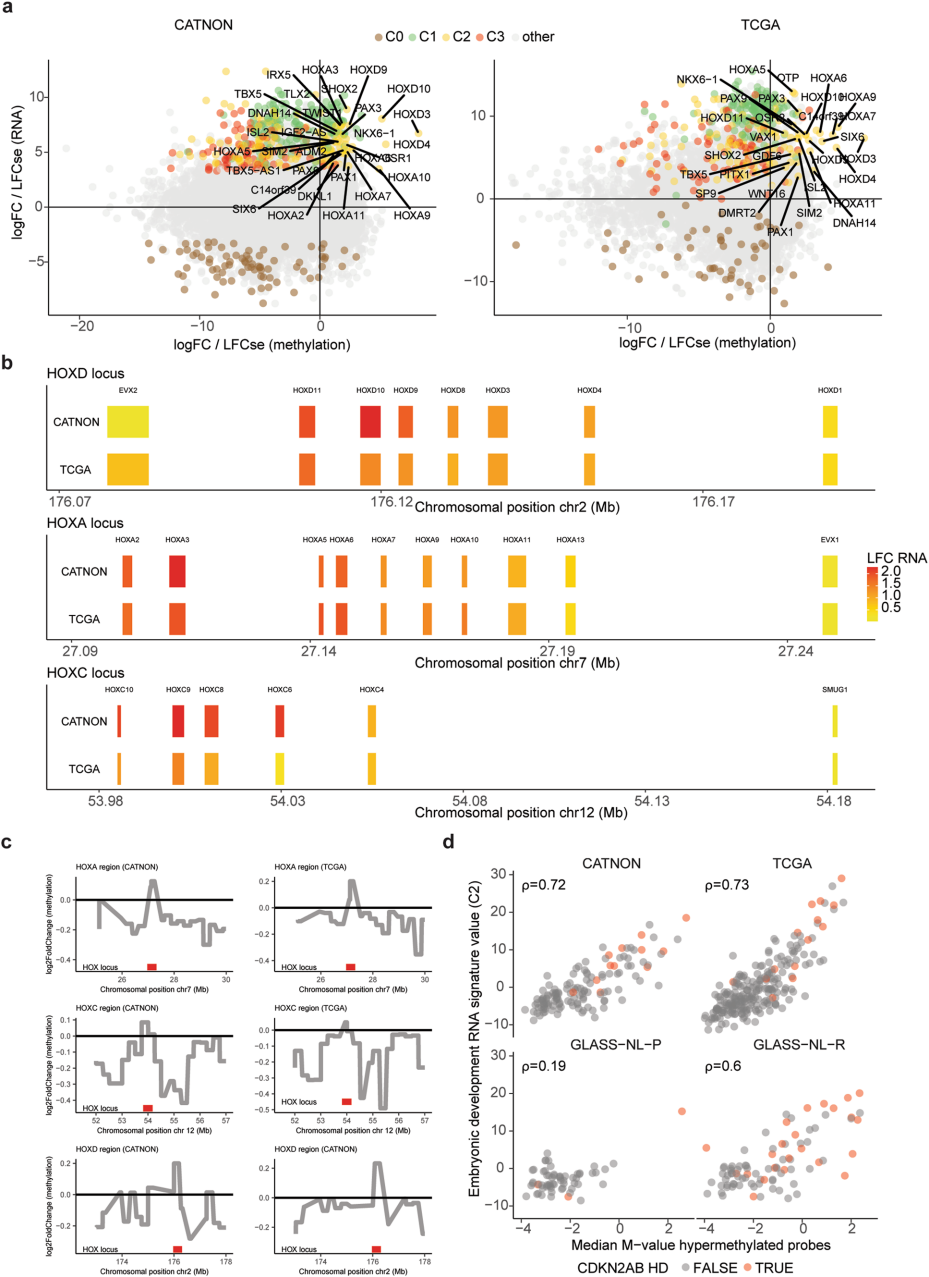
Supervised analysis reveals co-existence of upregulated and hypermethylated embryonic development genes.** a** Correlation between the DMP (x-axis) and DGR (y-axis) analyses across CATNON (850k chip) and TCGA (450k chip). **b** Log2FoldChange of the CGC on RNA VST expression across the *HOXD* (chr2), *HOXA* (chr7) and *HOXC* (chr12) loci. Chromosomal positions are indicated in megabases (Mb). **c** Log2FoldChange of the CGC on the DNAm *M*-values for the *HOX* loci (*HOXA, HOXC, and HOXD)* and the surrounding regions in CATNON (850k chip) and TCGA (450k chip). **d** Correlation between the embryonic development RNA signature scores (C2) and the median *M*-value of the hypermethylated probes. *CDKN2A/B* HD status is indicated in red

We next integrated the methylome differences with transcriptional changes of the corresponding genes (Fig. 5a). The differentially hypermethylated probes (*n* = 149) were associated with 63 genes from our RNA expression data (Supplementary Table 2). Pathway enrichment analysis on these hypermethylated genes revealed significant hits for high-CpG-density promoter (HCP) genes bearing the histone H3 trimethylation mark at K27 (H3K27Me3) in brain (FDR-adjusted *p* value = 4.8e−14).

Comparison of the hypermethylated genes with transcriptional changes of the corresponding genes revealed a strong overlap with the upregulated embryonic development cluster (C2, *n* = 27/63 genes). Thus, although the vast majority of the genes were hypomethylated with increased malignancy/CGC, we found hypermethylated CpGs to be associated with genes that were, paradoxically to what may be expected, transcriptionally upregulated. These hypermethylated and upregulated C2 genes encode for key developmental transcription factor genes, such as members of the *HOX* (*n* = 11), *PAX* (*n* = 2) and *TBX* (*n* = 1) family of genes. When we further examined the expression of the *HOX* genes of C2, we observed gene-ordered differential expression along the different gene clusters, where genes positioned at the 5’ end of the loci showed a higher expression with higher CGC scores and gradually decreased downstream in both CATNON and TCGA (Fig. 5b). This suggests coordinated derepression of enhancer sequences up/downstream of the gene cluster. Indeed, regions surrounding the *HOX* loci were hypomethylated, supporting the hypothesis of derepression of enhancers (Fig. 5c). Importantly, in both the bulk and single-cell data increased *HOX* expression of those present in C2 was not restricted to tumours with *CDKN2A/B* HD (Supplementary Fig. 5c).

The per-sample median *M*-value of the hypermethylated probes correlated strongly with the C2 signature score on both the CATNON (*ρ* = 0.72) and TCGA (*ρ* = 0.73) datasets (Fig. 5d). We also found a modest correlation in GLASS-NL-P (*ρ* = 0.19), which may be expected due to the low number of high-grade primary samples. Conversely, a strong correlation was evident GLASS-NL-R (*ρ* = 0.60) (Fig. 5d).

### Histopathological features associated with continuous grading coefficient

We investigated the correlation between thirteen histological features and the CGC through a multivariate linear model to gain insight into their association with tumour malignancy. For the CATNON dataset, thirteen histological features were scored by a panel of seven international neuropathologists: cell density, calcifications, increased number of blood vessels, microvascular proliferation, neoplastic-appearing astrocytes and oligodendrocytes, giant cells, (miniature) gemistocytes, nuclear pleomorphism, microcysts and mucoid degeneration, necrosis, and mitotic count [18]. Of these, the mitotic index (*p* = 0.0093) and giant cells (*p* = 0.042) were significantly associated with the CGC. This is in line with earlier work, which showed prognostic significance of the mitotic index [18]. We next correlated these variables with our RNA signatures and found that samples with a high mitotic index (> 2 mitoses per 10 40× consecutive high-power fields) and presence of giant cells had a higher C1 cell cycling (mitotic index: *p* = 0.015, giant cells: *p* = 0.029) and C2 embryonic development (mitotic index: *p* = 0.003, giant cells: *p* = 0.0019) signature value. In summary, our data revealed that increased cell cycling at the gene level is correlated with an elevated mitotic index.

### Hypermethylation phenotype and high expression of embryonic development genes are associated with poor survival

**Fig. 6:**
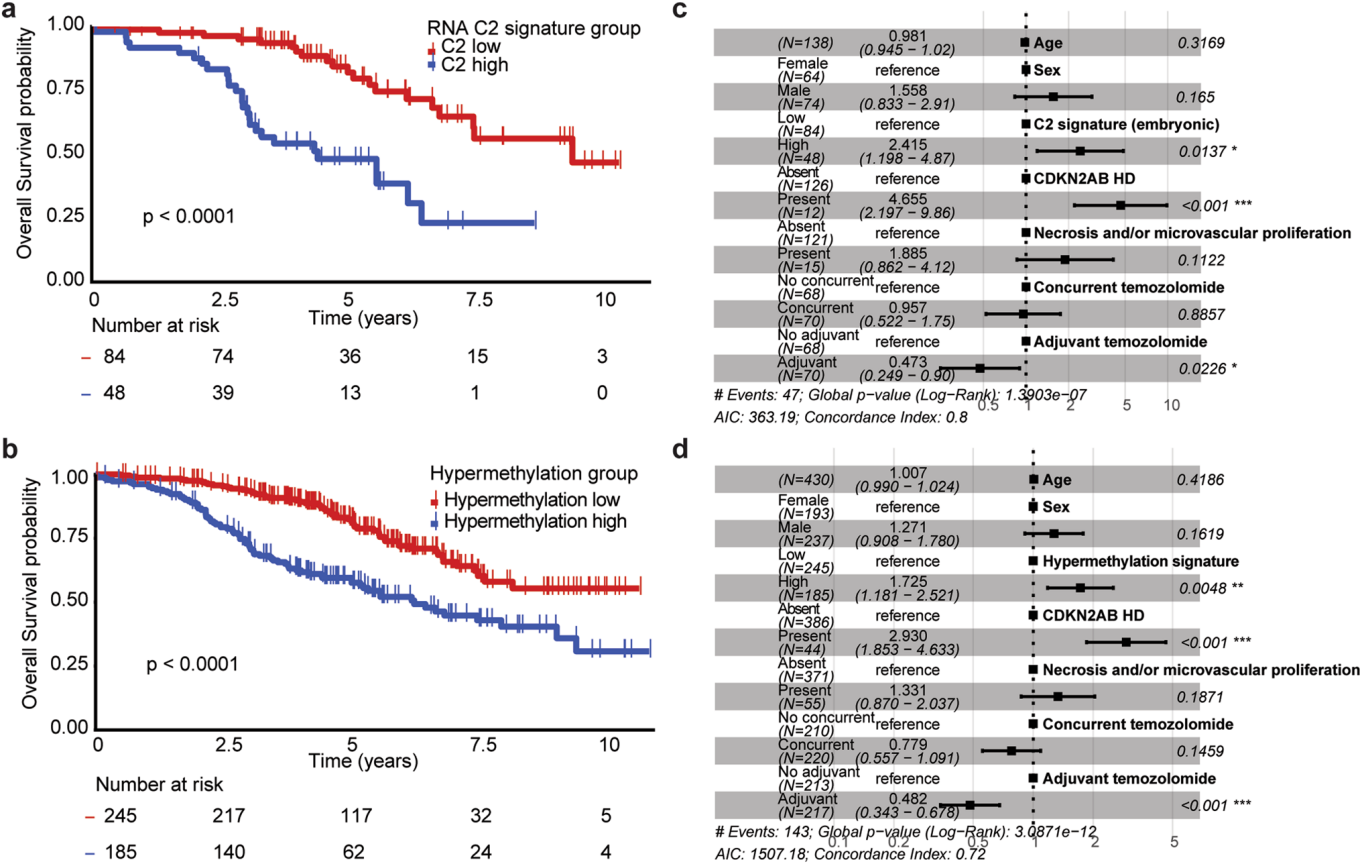
Survival analysis of CATNON based on RNA and methylation signatures. **a**/**c** Kaplan–Meier overall survival curves stratified by RNA C2 signature (**a**) and hypermethylation phenotype (**c**) risk groups. **b**/**d** Survival forest plots of predictive Cox proportional hazard models on the C2 signature (**b**) and hypermethylation phenotype (**d**) corrected for age, sex, *CDKN2A/B* HD, treatment and histology

We categorised samples into low- and high-risk groups for the C2 and hypermethylation signatures individually, based on the sign (negative or positive) of the C2 signature and PC1 associated with the *M*-value of hypermethylated probes (*n* = 149), respectively. Both high RNA expression of C2 genes (*p* < 0.0001, HR: 3.90 95% CI [2.13–7.13]) and the hypermethylation phenotype (*p* < 0.0001, HR: 2.42 95% CI [1.73–3.38]) were significantly associated with shorter survival in a univariate analysis on the CATNON dataset (Fig. 6a/b). These associations remained present after adjusting for age, sex, *CDKN2A/B* HD, microvascular proliferation/necrosis and adjuvant/concurrent temozolomide using a multivariable Cox PH model (C2 signature: *p* = 0.014, HR: 2.42 95% CI [1.20–4.87], hypermethylation phenotype: *p* = 0.0048, HR: 1.73 95% CI [1.18–2.52], Fig. 6c/d). These molecular signatures hold more prognostic value compared to the mitotic index (*p* = 0.053, HR: 1.66 95% CI [0.99–2.77]) in a univariate analysis.

The C2 RNA signature was associated with shorter survival in both TCGA (*p* < 0.0001, HR: 3.11 95% CI [1.7–5.6] and GLASS-NL-R (*p* = 0.0047, HR: 2.58 95% CI [1.30–5.11], Supplementary Fig. 9). The hypermethylation phenotype also showed prognostic significance in both TCGA (*p* < 0.0001, HR: 3.43 95% CI [1.93–6.11], and GLASS-NL-R (*p* = 0.012, HR: 1.92 95% CI [1.15–3.23], Supplementary Fig. 9).

## Discussion

In this study, we aimed to enhance the understanding of disparities in aggressiveness in IDH-mutant astrocytomas at a multi-domain molecular level. To do so, we generated RNA-sequencing data from IDH-mutant astrocytomas included in the CATNON randomised phase III clinical trial and combined it with additional multi-domain high-throughput data, along with multi-omics data from GLASS-NL and TCGA. We defined cut-off values for the methylation-based CGC for improved prognostication and found prognostic (epi)genetic and transcriptional markers that converge on three pathways: upregulation of cell-cycling genes, modification of the ECM, and tumour cell dedifferentiation (both by a reduced expression of mature astrocyte genes and upregulation of developmental genes). Importantly, altered expression of these three signatures was not restricted to tumours with *CDKN2A/B* HD, which demonstrates the additional prognostic power of our analyses. In recent years, the diagnostic classification of primary brain tumours has shifted from primarily histopathology to a more precision-oriented approach that incorporates molecular markers to optimise prognostication. We developed an objective measure, based on a DNAm-based classifier to assign a continuous grade per patient [6]. A shortcoming of the ‘CNS tumour classifier’ is that it only distinguishes between two epigenetic subgroups. We defined three astrocytoma subgroups based on the CGC, which adds an intermediate risk subgroup. These subclasses showed distinct OS, correlated with WHO CNS5 and high CNV load. Prognostic significance of these revised subgroups was further confirmed in our multivariable Cox regression analysis where they emerged as independent prognostic factor.

In all datasets, the CGC correlated strongly with the molecular marker *CDKN2A/B HD*, which is already incorporated in the WHO CNS5 classification. We also found members of the RB1 pathway to be individually associated with the CGC, including *CDK4* amplification and *RB1* HD. Alteration of these genes were described to correlate with malignancy in earlier work [2, 5, 38, 43]. Although these alterations were addressed in the cIMPACT-NOW update 5, evidence was not considered firmly established [5]. In addition, we identified *PDGFRA* amplification which has also been discussed as potential marker for grading [5]. These results support the CGC’s role as generic grading marker, since we used it to identify these associations, and it therefore encapsulates information on all these malignant genetic events. The CGC requires only a single assay to integrate multiple (rare) malignant alterations into the diagnostic system for which solid evidence may be hard provide. Also, continuity of tumour grading was supported since the incidence of these malignant events correlated with the value of the CGC. Our CGC grading model is easy to implement using the direct output from the ‘CNS tumour classifier’, but does require performing DNA-methylation arrays. One limitation of our study is that tumour location was not included in the data collection of the CATNON trial and therefore subgroup analyses based on this could not be performed.

After deducing transcriptional profiles associated with the CGC, we found multiple genes from the *HOX* gene clusters amongst our upregulated embryonic development genes. These genes exhibited a spatial sequential expression pattern reminiscent of that observed during embryogenesis. Despite genome-wide DNA-demethylation at progression, the *HOX* loci showed an increase in methylation levels and an increased RNA-expression. Hypermethylation of *HOX* genes has been described previously in glioma [4, 19, 20, 24]. However, there is no consensus regarding the subsequent epigenetic silencing or reactivation of these genes [4, 24]. We showed transcriptional upregulation and hypermethylation of the *HOXA*, *HOXC* and *HOXD* gene clusters in higher-grade IDH-mutant astrocytomas across multiple datasets. The ordered differential expression of *HOX* genes accompanied by focal and targeted hypermethylation, particularly located near the *HOX* genes, raises intriguing questions about their potential significance in malignant transformation. Biologically, hypermethylation of genes is often associated with gene repression [13]. Regulatory elements of *HOX* genes reside in topologically associating domains (TADs), which are involved in long-distance gene regulation [33]. In IDH-mutant gliomas, it has been shown that CTCF-binding is disrupted, leading to a loss of insulation between TADs and aberrant gene expression [10]. A recent study identified an increase in both H3K27Ac and H3K4Me3 peaks at known TSS of HOX and FOX transcription factors in IDH-mutant tumours with loss of DNA methylation, which is associated with their upregulation. This finding highlights a potential epigenetic activation mechanism in these tumours [23]. Importantly, our study was limited to correlative analyses and future experimental work on IDH-mutant glioma is needed to further explore a potential causal association between hypermethylation and transcriptional activation of embryonic development genes.

Besides providing an objective and clinically implementable means to grade IDH-mutant astrocytomas, our study is unique in that we used a prospectively collected sample cohort (CATNON), performed integrated analysis on three independent datasets, used treatment-naïve samples for signature development and included single-nucleus RNAseq data. These unique features allowed us to explore transcriptional associations and biological rationale underlying tumour malignancy. The CGC provides a comprehensive summary of the tumour’s malignant state and is not restricted to malignant events occurring in a small number of tumours but captures a broader spectrum of genomic alterations.

## Supplementary Information

Below is the link to the electronic supplementary material.

Supplementary file1 (DOCX 46 KB)

Supplementary file2 (PDF 33251 KB)

Supplementary file3 (XLSX 15 KB)

Supplementary file4 (XLSX 49 KB)

## Data Availability

The snRNA-seq data (vanHijfte_2023) generated in this study are publicly available in Zenodo (10.5281/zenodo.10408969). The raw bulk RNA-sequencing data have been deposited at the European Genome-Phenome Archive (EGA) and are publicly available (EGAD50000000558).
