# Supplementary material for: Epigenetic landscape reorganisation and reactivation of embryonic development genes are associated with malignancy in IDH-mutant astrocytoma
Source: Acta Neuropathol. 2024 Oct 9;148(1):50. doi: 10.1007/s00401-024-02811-0 (PMC11464554; doi:10.1007/s00401-024-02811-0)
Supplement: Supplementary file 1 — Supplementary file1 (DOCX 46 KB) [file 401_2024_2811_MOESM1_ESM.docx]

**Supplementary Methods**

**DNA methylation**

In total 751 patients were enrolled in the CATNON trial [2, 6]. DNA was isolated from FFPE tissue as described earlier [3]. After using the Infinium FFPE DNA Restoration Kit, DNA methylation profiling was performed with the Infinium MethlationEPIC BeadChip according to the manufacturer’s protocol. Patients were excluded from further analyses in case of insufficient material (n=78) or low-quality DNA methylation data (n=19). Low quality DNA methylation data was defined as >5% uninformative probes per sample at a detection P value < 0.01. 10 patients were classified as oligodendroglioma based on presence of 1p/19q codeletion and for 10 patients the *IDH1/2* status was unknown. 204 patients were excluded since tumours did not exhibit the *IDH1/2* mutation. The remaining 430 patients were included for this study.

Preprocessing of the raw idat files was performed using the minfi package [1]. Background noise correction and dye-bias normalisation were performed using the preprocessNoob function. Poor performing probes with a detection P value >.01 were omitted (CATNON: n=67,849, GLASS-NL: n=91,130, TCGA: n=38,435). Also, probes previously described to underperform were excluded [9] (Infinium EPIC array: n=105,454, Infinium 450K array: n=92,783).

**RNA-sequencing**
For the CATNON dataset, Illumina sequencing reads were obtained as compressed fastq files (n=183 samples). Unique Molecular Identifiers (UMI) were extracted to the read header using UMI-tools (v.1.2.2). Using fastp (v.0.23.2), low complexity reads were filtered, polyG reads longer than 35 bases were trimmed and unwanted polyX (i.e. PolyA) tailing was removed. After quality control with both fastp and fastQC (v.0.23.2), reads were mapped to the human reference genome (hg19/GRCh37) using the STAR aligner (v.2.7.9a). The minimum overhang for annotated and unannotated junctions were set to 1 and 10 bases respectively. Expected intron lengths were set between 20 and 1000000 bases. Maximum genomic distance between mates was set to 1000000. The minimum chimeric segment length and overhang for a chimeric junction were set to 12. Alignments that contained non-canonical unannotated junctions were filtered. Resulting bam files were sorted and indexed using SAMtools (v.1.9). UMI were deduplicated using umi tools whereby chimeric read pairs and unpaired reads were discarded. The template length was ignored for paired-end deduplication. The number of reads mapped to each chromosome was determined using SAMtools IdxStats. FeatureCounts (v.2.0.3) was used to count the number of reads for each corresponding gene using gencode v34 as gene annotation. The mean number of assigned reads was ~8.5 million. Samples with FeatureCounts assigned read counts below 500,000 (n=28) were excluded. In case of duplicates (n=2) the sample with the highest number of assigned reads was selected. For samples with assigned read counts between 500,000 and 750,000 we excluded samples with a high percentage of reads mapping to alternate loci (>50%, n=2) and outliers in an unsupervised PCA (n=1). Two PCA outliers with more than 750,000 reads were excluded due to a bad score in multiple other QC metrics (alternate loci mapping, GC content and UMI count). 148 samples passed our quality control pipeline, from which we excluded re-resections (n=8) and samples without matching methylation data (n=2). Sequencing protocols and processing of read counts for the GLASS-NL dataset are described in an earlier study [7].

**Tumour purity estimation**

We evaluated two tumour purity estimation methods by their correlation with each other and with RNA expression levels of genes associated with neurons. One method was based on DNA Whole Exome Sequencing (WES) data to subsequently estimate tumour purity by the Variant Allele Frequency (VAF) of the IDH1/2 mutation. We hereby assumed no loss of heterozygosity and that the IDH1/2 mutation was a clonal mutation. The R-package InfiniumPurify estimates tumour purity by using informative differentially methylated CpG sites (iDMC) between tumour samples and a reference set of normal brain samples [5]. The RNA expression levels per cell type were calculated by selecting the top 100 marker genes provided by McKenzie et al [4]. This information was summarised to a single component per cell type, by using principal component 1 (PC1) from the output of Principal Component Analysis (PCA) on the marker genes per cell type.

**RNA-sequencing analysis of purified human CNS cell types**

We acquired transcriptome profiles (n=23,223 genes) of purified human CNS cell types from the original publication [8]. In short, foetal astrocytes (n=6) were isolated from brain tissue samples obtained during elective pregnancy terminations, while mature astrocytes (n=12) were derived from juvenile and adult brain tissue samples (8y-63y) acquired during neurological surgeries. Oligodendrocytes (n=5) and tumour-associated macrophages/microglia (n=3) were isolated from adult brain tissue samples. We compared the mean Fragments Per Kilobase Million (FPKM) values for our downregulated (C0) and upregulated (C1-C3) transcriptional clusters between cell types.

1. Aryee MJ, Jaffe AE, Corrada-Bravo H, Ladd-Acosta C, Feinberg AP, Hansen KD, Irizarry RA (2014) Minfi: a flexible and comprehensive Bioconductor package for the analysis of Infinium DNA methylation microarrays. Bioinformatics 30:1363–1369. https://doi.org/10.1093/BIOINFORMATICS/BTU049

2. van den Bent MJ, Baumert B, Erridge SC, Vogelbaum MA, Nowak AK, Sanson M, Brandes AA, Clement PM, Baurain JF, Mason WP, Wheeler H, Chinot OL, Gill S, Griffin M, Brachman DG, Taal W, Rudà R, Weller M, McBain C, Reijneveld J, Enting RH, Weber DC, Lesimple T, Clenton S, Gijtenbeek A, Pascoe S, Herrlinger U, Hau P, Dhermain F, van Heuvel I, Stupp R, Aldape K, Jenkins RB, Dubbink HJ, Dinjens WNM, Wesseling P, Nuyens S, Golfinopoulos V, Gorlia T, Wick W, Kros JM (2017) Interim results from the CATNON trial (EORTC study 26053-22054) of treatment with concurrent and adjuvant temozolomide for 1p/19q non-co-deleted anaplastic glioma: a phase 3, randomised, open-label intergroup study. Lancet 390:1645–1653. https://doi.org/10.1016/S0140-6736(17)31442-3

3. Draaisma K, Chatzipli A, Taphoorn M, Kerkhof M, Weyerbrock A, Sanson M, Hoeben A, Lukacova S, Lombardi G, Leenstra S, Hanse M, Fleischeuer R, Watts C, McAbee J, Angelopoulos N, Gorlia T, Golfinopoulos V, Kros JM, Verhaak RGW, Bours V, van den Bent MJ, McDermott U, Robe PA, French PJ (2020) Molecular evolution of IDH wild-type glioblastomas treated with standard of care affects survival and design of precision medicine trials: A report from the EORTC 1542 study. J Clin Oncol 38:81–99. https://doi.org/10.1200/JCO.19.00367

4. McKenzie AT, Wang M, Hauberg ME, Fullard JF, Kozlenkov A, Keenan A, Hurd YL, Dracheva S, Casaccia P, Roussos P, Zhang B (2018) Brain Cell Type Specific Gene Expression and Co-expression Network Architectures. Sci Reports 2018 81 8:1–19. https://doi.org/10.1038/s41598-018-27293-5

5. Qin Y, Feng H, Chen M, Wu H, Zheng X (2018) InfiniumPurify: An R package for estimating and accounting for tumour purity in cancer methylation research. Genes Dis 5:43–45. https://doi.org/10.1016/J.GENDIS.2018.02.003

6. Tesileanu CMS, Van Den Bent MJ, Sanson M, Wick W, Brandes AA, Clement PM, Erridge SC, Vogelbaum MA, Nowak AK, Baurain JF, Mason WP, Wheeler H, Chinot OL, Gill S, Griffin M, Rogers L, Taal W, Rudà R, Weller M, McBain C, Van Linde ME, Sabedot TS, Hoogstrate Y, Von Deimling A, De Heer I, Van Ijcken WFJ, Brouwer RWW, Aldape K, Jenkins RB, Dubbink HJ, Kros JM, Wesseling P, Cheung KJ, Golfinopoulos V, Baumert BG, Gorlia T, Noushmehr H, French PJ (2021) Prognostic significance of genome-wide DNA methylation profiles within the randomized, phase 3, EORTC CATNON trial on non-1p/19q deleted anaplastic glioma. Neuro Oncol 23:1547–1559. https://doi.org/10.1093/NEUONC/NOAB088

7. Vallentgoed WR, Hoogstrate Y, van Garderen KA, van Hijfte L, van Dijk E, Kouwenhoven MCM, Niers A, Westerman B, Wesseling P, French PJ (2024) Evolutionary trajectories of IDH-mutant astrocytoma identify molecular grading markers related to cell cycling [Submitted]

8. Zhang Y, Sloan SA, Clarke LE, Caneda C, Plaza CA, Blumenthal PD, Vogel H, Steinberg GK, Edwards MSB, Li G, Duncan JA, Cheshier SH, Shuer LM, Chang EF, Grant GA, Gephart MGH, Barres BA (2016) Purification and Characterization of Progenitor and Mature Human Astrocytes Reveals Transcriptional and Functional Differences with Mouse. Neuron 89:37–53. https://doi.org/10.1016/J.NEURON.2015.11.013

9. Zhou W, Laird PW, Shen H (2017) Comprehensive characterization, annotation and innovative use of Infinium DNA methylation BeadChip probes. Nucleic Acids Res 45:e22–e22. https://doi.org/10.1093/NAR/GKW967
