# Supplementary material for: Epigenetic landscape reorganisation and reactivation of embryonic development genes are associated with malignancy in IDH-mutant astrocytoma
Source: Acta Neuropathol. 2024 Oct 9;148(1):50. doi: 10.1007/s00401-024-02811-0 (PMC11464554; doi:10.1007/s00401-024-02811-0)
Supplement: Supplementary file 2 — Supplementary file2 (PDF 33251 KB) [file 401_2024_2811_MOESM2_ESM.pdf]

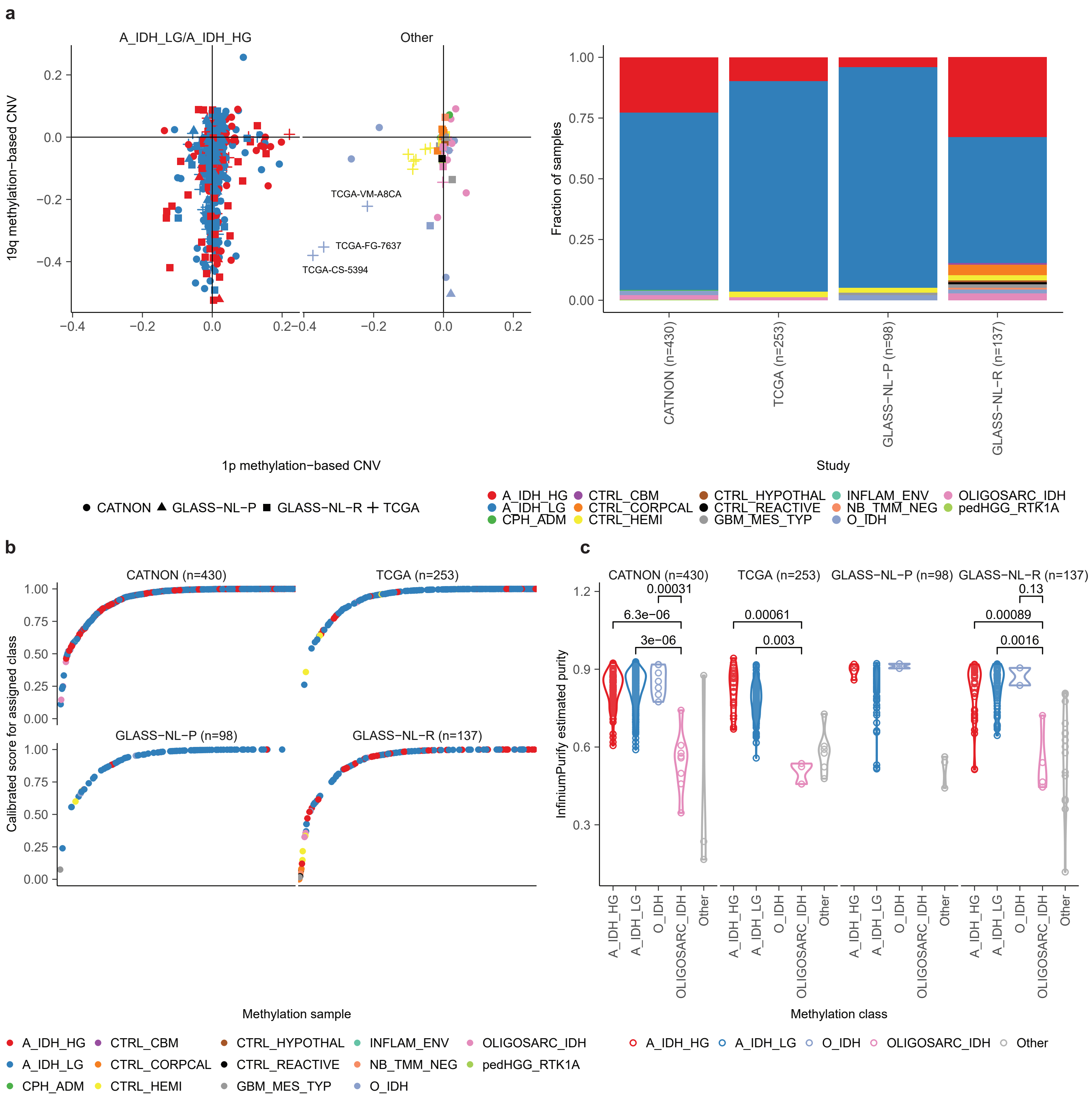

Supplementary Figure 1 Assignment of CNS tumour subtypes by the CNS tumour classifier and assessment of tumour purity for the CATNON (850k chip), TCGA (450k chip), GLASS-NL-P (850k chip) and GLASS-NL-R (850k chip) datasets. **a** Methylation-based copy number variation estimates for chr1p and chr19q (left) and subtype classification (right). **b** Calibrated scores for the assigned subtype classes per dataset. **c** Estimated purities for the assigned methylation classes. P-values determined by Wilcoxon signed-rank test.

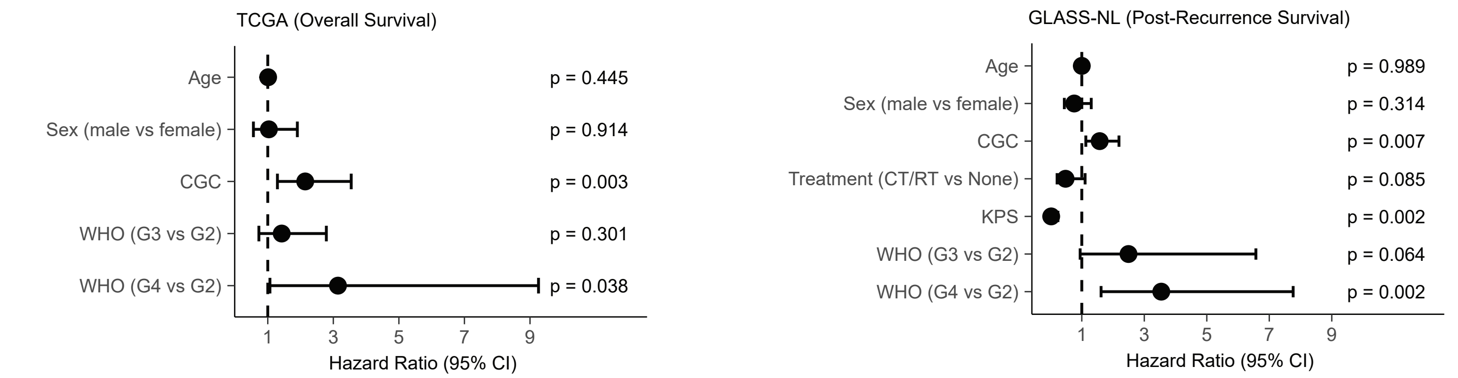

Supplementary Figure 2 Survival forest plots depicting predictive Cox proportional hazard models for TCGA (left) and GLASS-NL (right), adjusted for age, sex, Continuous Grading Coefficient (CGC) and WHO CNS5. The GLASS-NL model is additionally adjusted for Karnofsky Performance Score (KPS) and treatment (CT: chemotherapy, RT: radiotherapy).

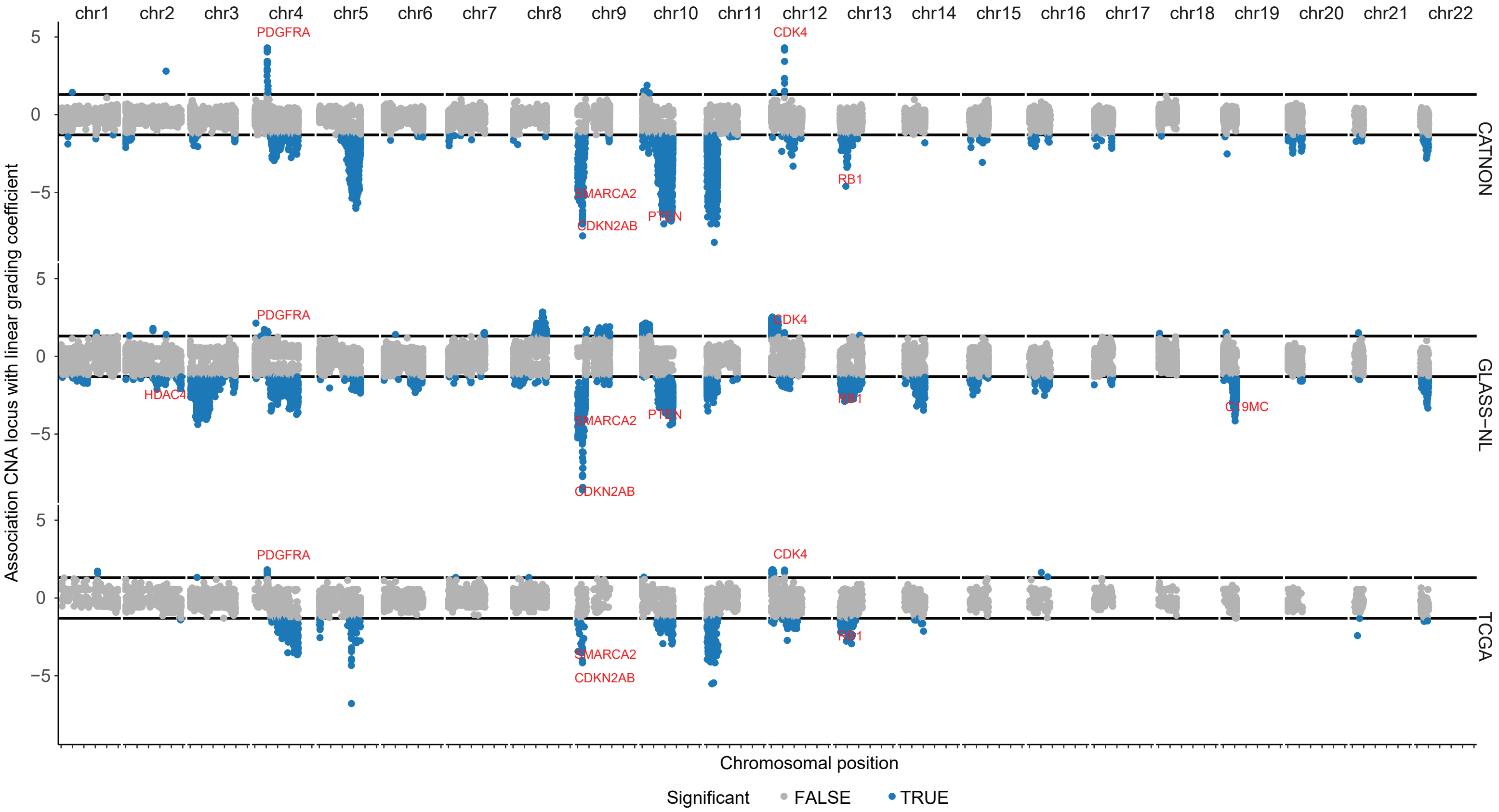

Supplementary Figure 3 Associations (log10(FDR-adjusted p-value)) between the CNA locus and the Continuous Grading Coefficient (CGC) for the CATNON (850k chip), TCGA (450k chip) and GLASS-NL (850k chip) datasets. P-values were determined by the Wilcoxon signed-rank test.

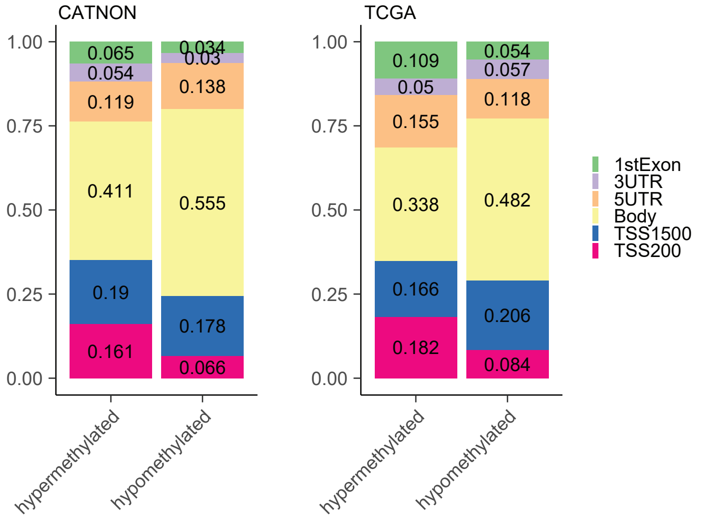

Supplementary Figure 4 Distribution of probes belonging to 1st exon region, 3’UTR, 5’UTR, gene body and TSS200/1500 across all hypomethylated and hypermethylated probes. Distributions are displayed separately for the DMPs resulting from independent analyses conducted on CATNON (850k chip, left) and TCGA (450k chip, right).

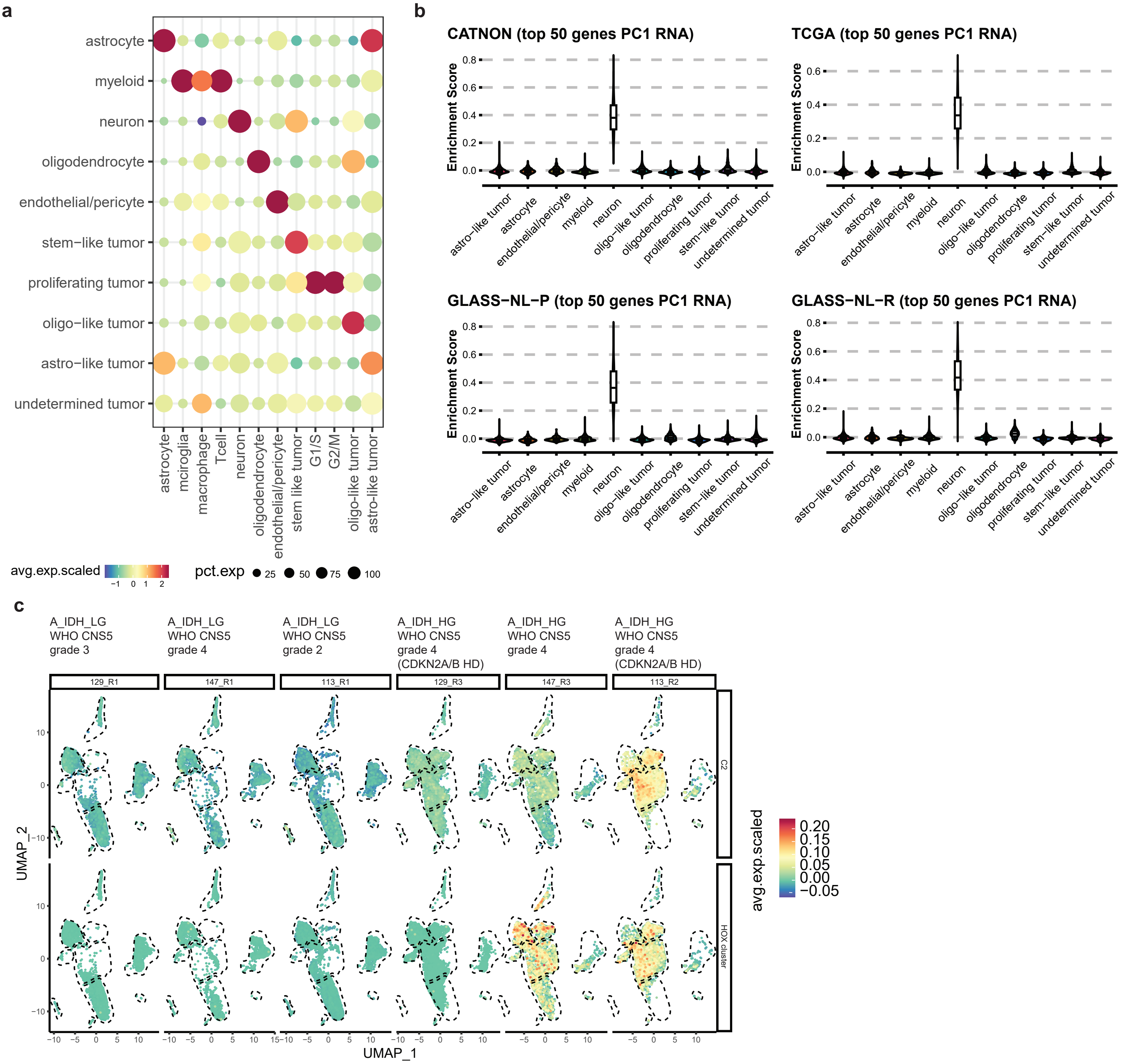

Supplementary Fig. 5 Single-nucleus RNA sequencing enrichment scores on methylation-based low-grade and high-grade samples. **a** Dotplot showing the enrichment scores of marker genes for each of the assigned cell types. **b** Violin plot illustrating enrichment scores per cell-type for the top 50 most contributing genes from the unsupervised PCA on CATNON, TCGA, GLASS-NL-P and GLASS-NL-R. **c** UMAP projections showing enrichment scores of the embryonic development (C2) cluster and HOX genes (from C2). CDKN2A/B HD status is indicated.

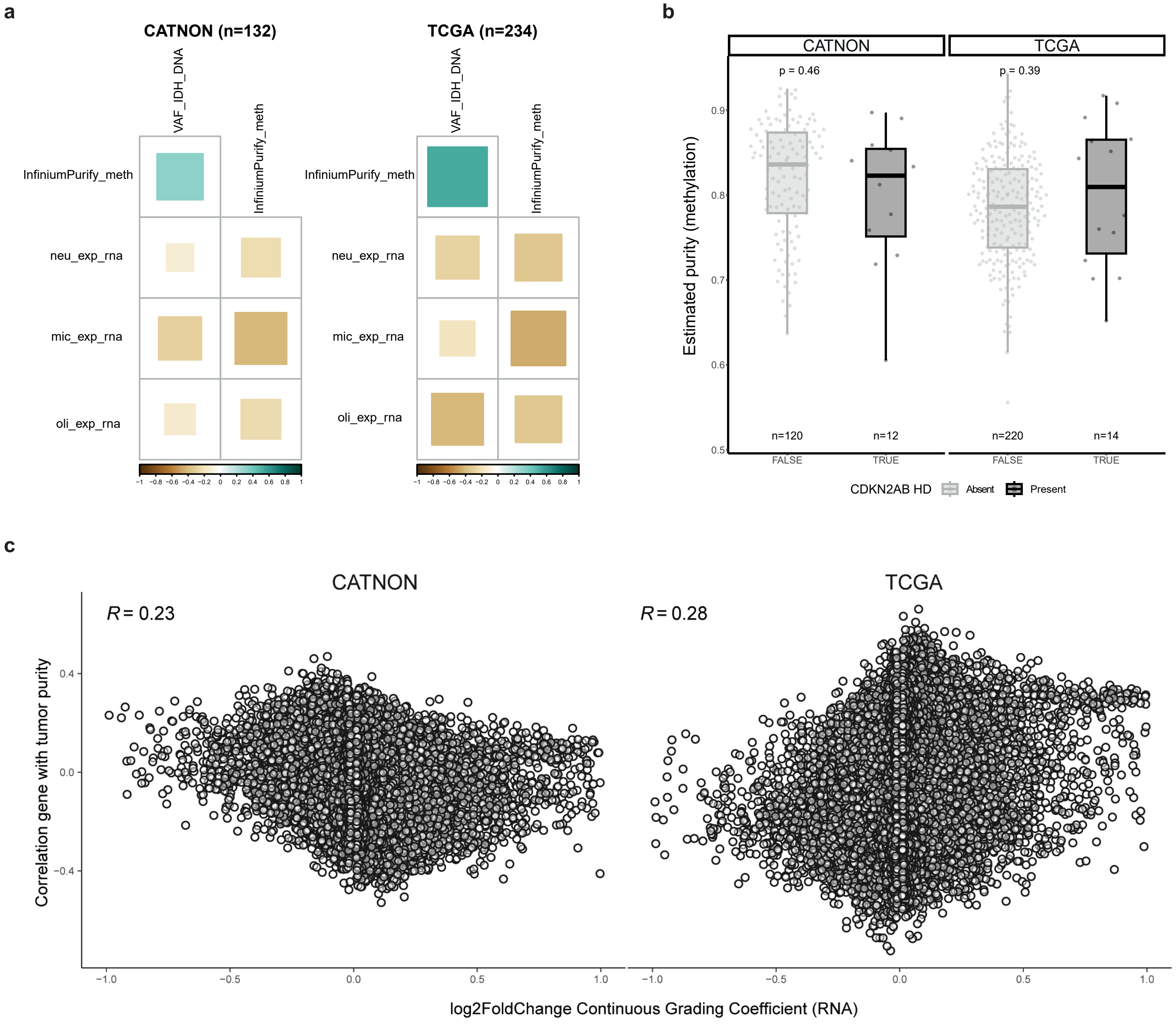

Supplementary Fig. 6 Association between tumour purity and outcome of the DGR analysis. **a** Correlation between the expression of oligodendrocyte, microglia and neuron expression marker genes and tumour purity estimation methods (VAF IDH mutation, InfiniumPurify) for both the CATNON (850k) and TCGA (450k) datasets. **b** Distribution of InfiniumPurify estimated purities based on the presence of CDKN2A/B HD. For both the CATNON and TCGA dataset no significant difference in tumour purity was observed. P-values determined by Wilcoxon signed-rank test. **c** Correlation of the log2FoldChange (RNA) of the Continuous Grading Coefficient (CGC) with InfiniumPurify estimated purities.

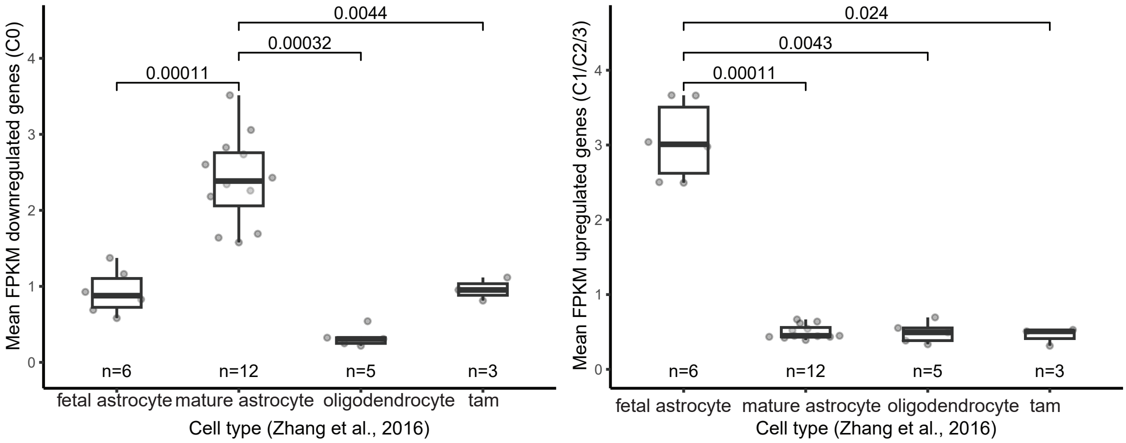

Supplementary Fig. 7 Mean FPKM of downregulated (C0, left) and upregulated (C1/C2/C3) transcriptional cluster genes identified in our DGR analysis across fetal astrocytes (n=6), mature astrocytes, oligodendrocytes and tumour-associated macrophages. P-values determined by Wilcoxon signed-rank test.

a

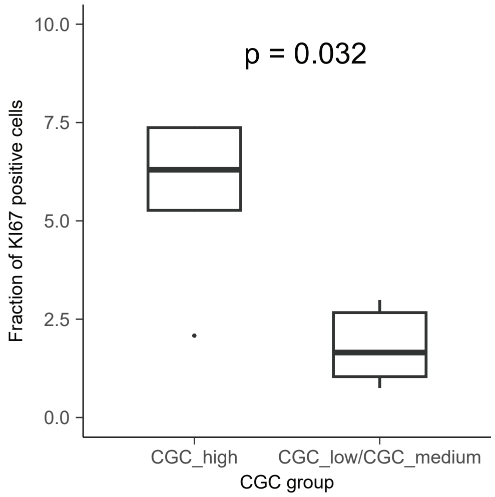

b

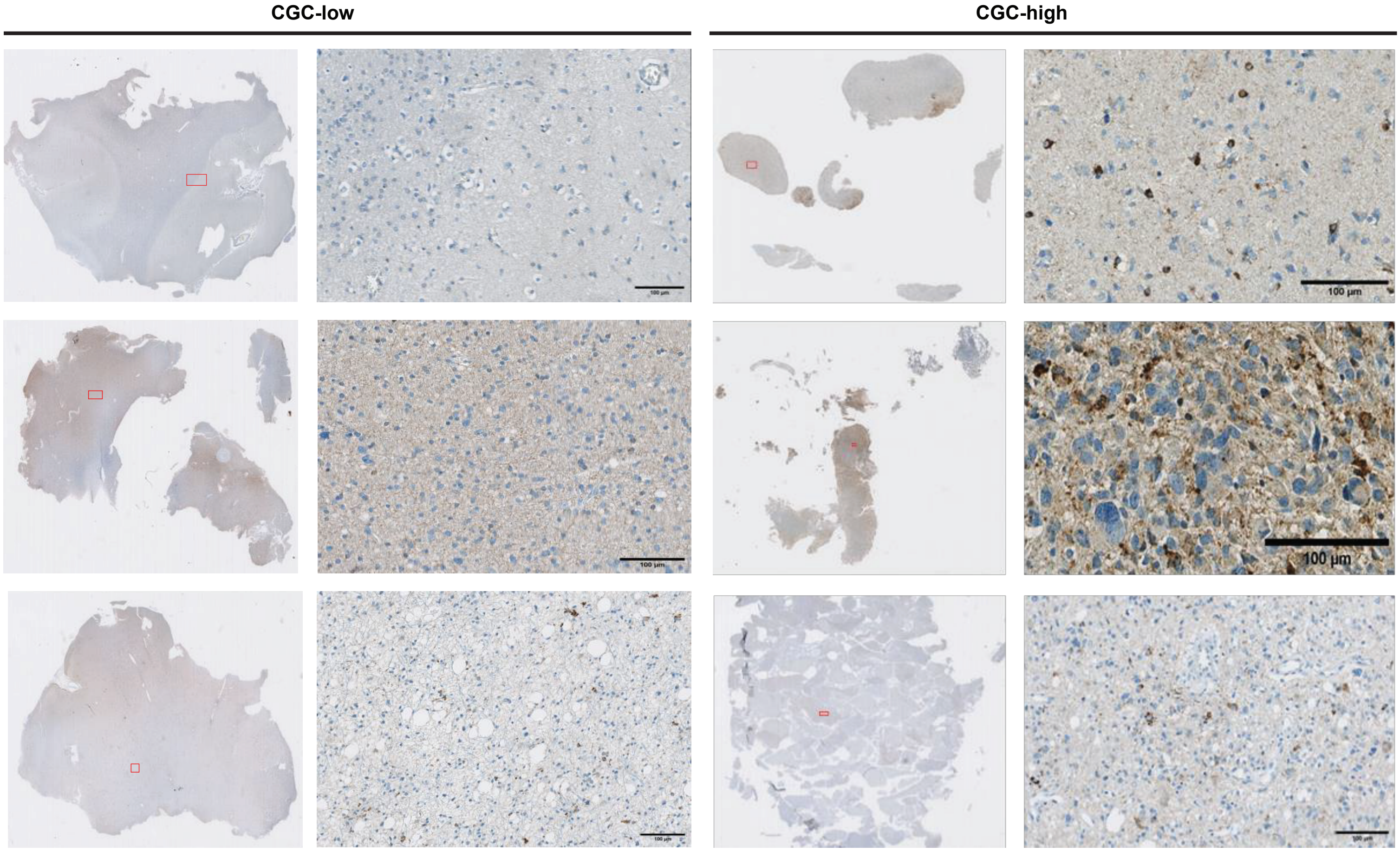

Supplementary Fig. 8 Immunohistochemical analysis of KI67 and HOXD10 expression using DAB staining **a** Whole-slide quantification of KI67-positive cells in CGC-low/medium (n=5) and CGC-high (n=5) samples. Statistical significance was determined using the Wilcoxon test ($p<0.05$) **b** Representative images showing HOXD10 expression in CGC-low (n=3) and CGC-high (n=3) samples. Scale bar = 100 µm.

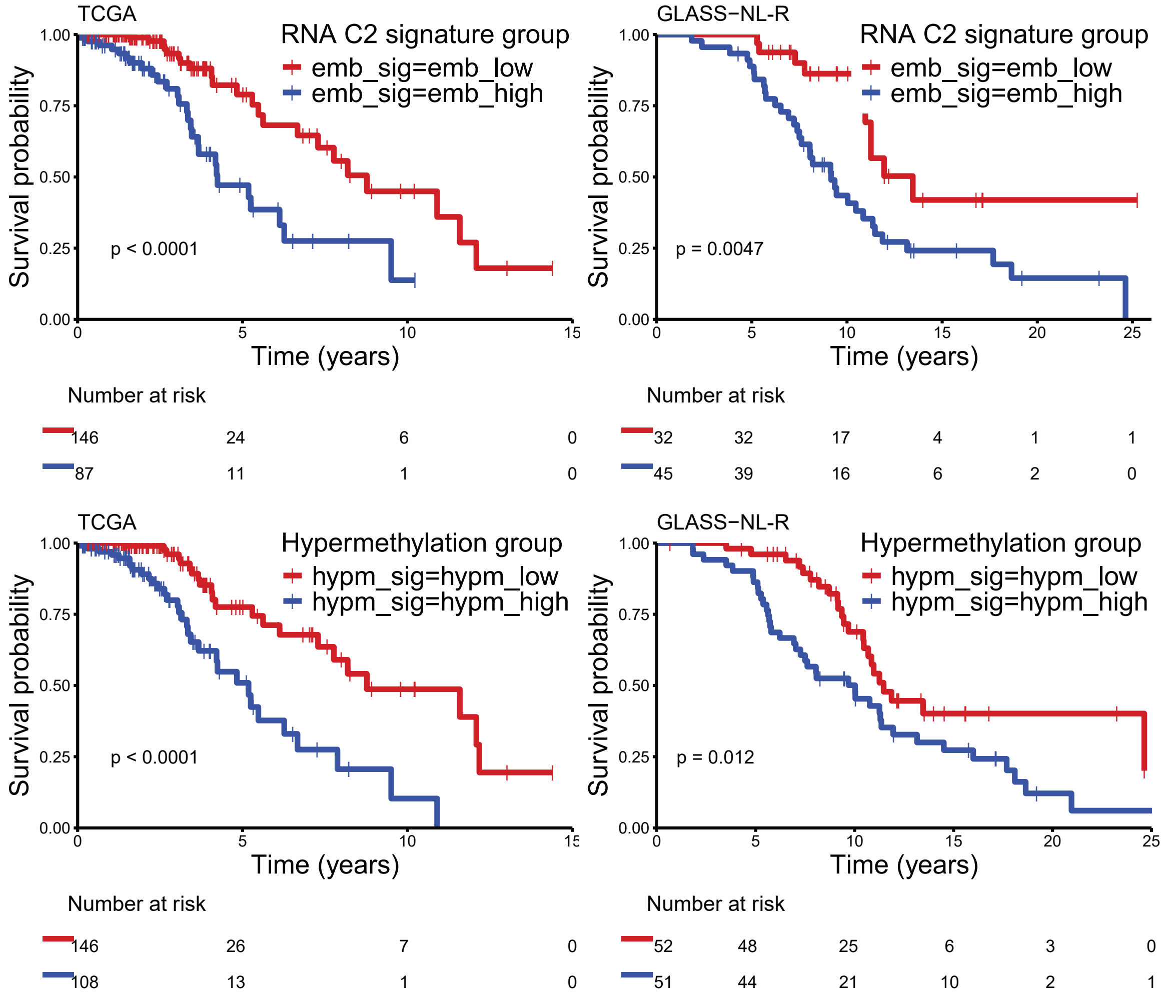

Supplementary Fig. 9 Kaplan-Meier overall survival curves of TCGA and GLASS-NL-R stratified by RNA C2 signature (top) and hypermethylation risk (bottom) groups. Log-rank test was used to determine significance.
